# Non-equilibrium anisotropic colloidal single crystal growth with DNA

**DOI:** 10.1038/s41467-018-06982-9

**Published:** 2018-11-01

**Authors:** Soyoung E. Seo, Martin Girard, Monica Olvera de la Cruz, Chad A. Mirkin

**Affiliations:** 1 Department of Chemistry, Northwestern University, Evanston, IL 60208 USA; 2 International Institute for Nanotechnology, Northwestern University, Evanston, IL 60208 USA; 3 Department of Materials Science and Engineering, Northwestern University, Evanston, IL 60208 USA; 4 Department of Physics and Astronomy, Northwestern University, Evanston, IL 60208 USA

## Abstract

Anisotropic colloidal crystals are materials with novel optical and electronic properties. However, experimental observations of colloidal single crystals have been limited to relatively isotropic habits. Here, we show DNA-mediated crystallization of two types of nanoparticles with different hydrodynamic radii that form highly anisotropic, hexagonal prism microcrystals with AB_2_ crystallographic symmetry. The DNA directs the nanoparticles to assemble into a non-equilibrium crystal shape that is enclosed by the highest surface energy facets (AB_2_(10$\overline 1$0) and AB_2_(0001)). Simulations and theoretical arguments show that this observation is a consequence of large energy barriers between different terminations of the AB_2_(10$\overline 1$0) facet, which results in a significant deceleration of the (10$\overline 1$0) facet growth rate. In addition to reporting a hexagonal colloidal crystal habit, this work introduces a potentially general plane multiplicity mechanism for growing non-equilibrium crystal shapes, an advance that will be useful for designing colloidal crystal habits with important applications in both optics and photocatalysis.

## Abstract (web summary)

Colloidal crystal engineering with DNA can be used to synthesize highly anisotropic hexagonal prismatic microcrystals. This manuscript introduces a plane multiplicity mechanism that can be used to deliberately design non-equilibrium Wulff shapes, a capability important in many areas, including optics and photocatalysis.

## Introduction

The crystal shape (habit) of atomic systems is an external expression of internal structure. The shape that a set of atoms adopts is largely dictated by the configurations of valence electrons unique to each atom and crystallization conditions. In general, an equilibrium shape emerges from the anisotropy of the surface energy, where preferential growth of the lowest surface energy facet is thermodynamically favored^1,2^. Various factors (e.g., pressure, temperature, concentration) can be used to generate single crystals with different structures and properties^3^, which inform their use in a variety of applications, including solar cells^4,5^ and optical devices^6,7^. For colloidal crystals, recent a priori findings on the properties of three-dimensional (3D) microcrystals composed of inorganic nanoparticles suggest that the use of shape anisotropy is a promising path to designing and controlling optical responses in metamaterials^8–10^. In particular, nano- and microstructures with hexagonal cross-sections (e.g., hexagonal wires), which occur naturally in GaN, are interesting because such structures support whispering gallery modes that exhibit low power loss and high-quality factors^11,12^. Furthermore, these crystals can be used for nonlinear optics^13^, an area where nanocomposites are promising due to enhancement of nonlinear susceptibility^14^, or for photocatalysis^15^. However, despite significant interest in emergent properties associated with such structures, this is, as of yet, done empirically because little is known about ways to control mesoscale crystal shape and morphology. Although atomic crystals with unique geometric features have been synthesized by kinetically controlling growth, kinetic structures in colloidal crystal engineering have been avoided because they are difficult to deliberately make and often have many defects. There are experimental studies reporting unique optical properties of noble metal nanoparticle-based metamaterials. However, these materials are produced through drying and sedimentation techniques that prevent the controllable formation of specific and desired crystal habits, crystal symmetries, and lattice parameters^16–18^.

To this end, the use of programmable atom equivalents (PAEs) generated from nanoparticles and synthetically tunable DNA ligand shells is a promising route for making ordered, crystalline structures with diverse crystallographic symmetries in a manner that is analogous to atomic crystallization^19,20^. Here, particle interactions driven by the DNA bond afford deliberate architectural control with tunable bond length and strength^13,21–24^. Indeed, one of the attractive features of this approach is that design rules have been established that allow one to control crystal symmetry, lattice parameter, and habit, independent of particle composition but dependent on particle size and shape^25–27^. Using a slow cooling approach, high-quality 3D single crystals can be produced^28,29^. This strategy has been used to prepare several types of single-crystalline architectures, including rhombic dodecahedra, cubes, and octahedra^28,29.^ Importantly, all single-crystal systems studied thus far have been limited to structures with cubic symmetry, and therefore the high symmetry internal packing provides access to only a small number of isotropic crystalline shapes.

Experimental observations of single crystals have been restricted to PAEs with similar hydrodynamic radii (a combination of the nanoparticle radius and the DNA length). Here, we show that combining nanoparticles of different sizes can alter the interface kinetics in the system, leading to the formation of highly anisotropic single crystals. The AB_2_ (isostructural with the intermetallic phase aluminum diboride) structure yields elongated hexagonal prism microcrystals upon slow cooling. Importantly, these anisotropic microcrystals can be realized from the AB_2_ structure with different lattice parameters. The experimental results are corroborated with theoretical studies and suggest that the formation of highly anisotropic single crystals is facilitated by anisotropy in the interface kinetics, where the fast-growing facets grow out and disappear, and the slow-growing facets, AB_2_(10$\overline 1$0) and AB_2_(0001) (the facet normal to AB_2_(10$\overline 1$0)), are present in the final shape. It is important to note that based on molecular dynamics (MD) simulation results, the facets enclosing the crystals have the highest surface energies, in contrast with usual equilibrium Wulff constructions. Such crystal growth behavior stems from the nucleation barrier between each termination of AB_2_(10$\overline 1$0), which significantly decelerates the growth rate due to the energetic penalty involved in nucleation of a layer with a high surface energy atop a layer with a low surface energy. The results shown in this work present a solution to a synthetic and materials challenge in colloidal crystal engineering that had not been addressed previously, where the deviation from minimal energy shapes and the broken bond model picture enables the synthesis of anisotropic colloidal crystals. This further suggests that external parameters, such as ionic strength, temperature, and stress, could be used to control colloidal crystallization. The ability to design and generate colloidal crystals with desired crystal shape is crucial for the fabrication of next-generation optoelectronic devices since optical modes are highly dependent on crystal shape.

## Results

### DNA-programmable assembly of anisotropic colloidal crystals

In order to realize colloidal crystals with anisotropic shapes, three main principles were identified: (i) the combination of two types of particles should form lattices with a crystallographic symmetry other than cubic (e.g., hexagonal single crystals can only be generated from hexagonal lattices), (ii) the selected structure should have no competing structures to minimize defects which will inhibit single-crystal growth, and (iii) the difference in growth rates of different planes should be large enough to favor the dominant growth of a specific surface facet(s) and, therefore, one preferred type of single crystal. Colloidal crystal engineering with DNA represents an ideal means to control the internal particle packing because the assembly behavior of PAEs is well-understood based upon the complementary contact model^19^ and potential energy calculations^30^. PAEs enable one to deliberately and independently toggle nanoparticle size, composition, bond strength, and interparticle spacing, while maintaining a desired crystal symmetry in a controllable fashion. Specifically, the ratio between the hydrodynamic radii of the building blocks was used as a design parameter to produce the microcrystals presented in this work. To test our principles for the assembly of highly anisotropic microcrystals, superlattices with crystallographic symmetry identical to AlB_2_ were chosen as a model system since this structure meets our criteria.

**Fig. 1:**
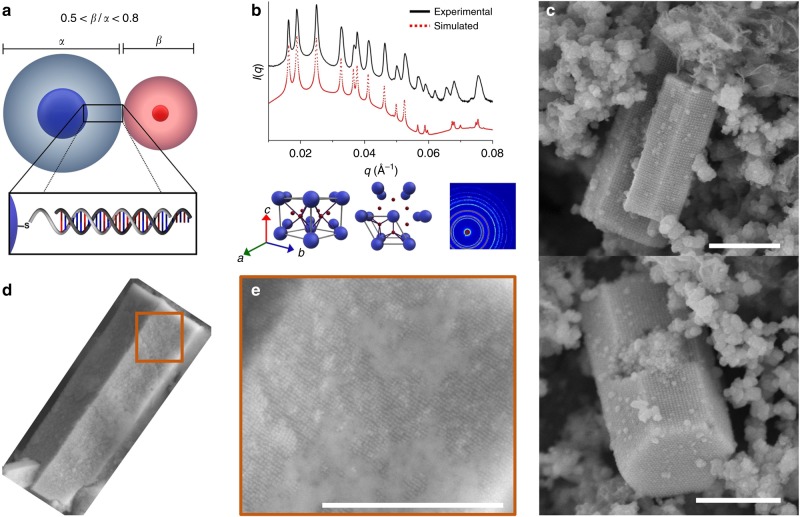
Hexagonal prism microcrystals formed from DNA-functionalized nanoparticles. **a** Scheme of a set of DNA-functionalized gold nanoparticles with a hydrodynamic radius ratio (*β*/*α*) between 0.5 and 0.8 used to produce superlattices with AB_2_ crystallographic symmetry. *α* and *β* denote hydrodynamic radii of particles A and B. **b** Representative one-dimensional (top) and two-dimensional (bottom, right) SAXS data for AB_2_ superlattices assembled from the slow cooling approach. Different views of the AB_2_ unit cell (bottom, left) are shown for reference. SAXS data are shown as plots of the overall scattering intensity from a crystal domain (*I(q)*, in arbitrary units) versus scattering vector (*q*, in units of Å^−1^). **c** SEM images of hexagonal prism microcrystals in different orientations. **d**, **e** SEM images of a representative microcrystal with visible faceting (**d**) where constituent nanoparticles can be seen (**e**). Scale bars are 1 μm

In order to make colloidal crystals with AB_2_ symmetry, pairs of PAEs (with hydrodynamic radius ratios between 0.5 and 0.8) were synthesized based upon the previously established colloidal crystal engineering design rules^19^. The slow cooling of two sets of PAEs with complementary DNA linker strands produces microcrystals with the AB_2_ packing when the hydrodynamic radius ratio (β/α) of the PAEs falls within this range (Fig. 1a). The hydrodynamic radius of each PAE was determined by calculating the DNA length and adding it to the core radius (see the equation in Hydrodynamic Radius Calculation section of Supplementary Information). The internal ordering of the PAE superlattices was confirmed by 1D radial line averages of the small angle X-ray scattering (SAXS) patterns (Fig. 1b). The surface features and the overall crystal shape were determined by scanning electron microscopy (SEM), where images of the hexagonal prismatic microcrystals reveal uniform single crystals defined by six rectangular-shaped faces and two hexagonal-shaped bases (Fig. 1c, d). In addition, high-resolution SEM images show extraordinarily well-formed crystals with smooth crystal facets, consisting of highly ordered nanoparticles (Fig. 1e).

### Tunable structural parameters at the nanoscale

**Fig. 2:**
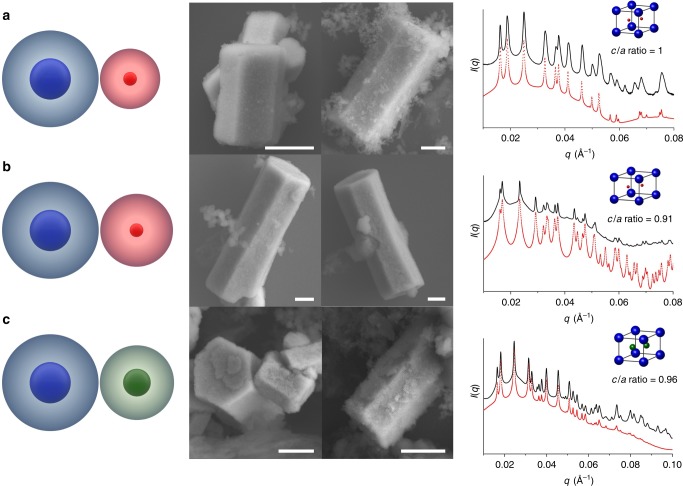
Hexagonal prisms with varying unit cell compositions. **a**–**c** SEM images and one-dimensional SAXS patterns of hexagonal prism microcrystals from an AB_2_ lattice synthesized from different sets of PAEs to achieve a range of *β*/*α*: **a** 0.67, **b** 0.73, and **c** 0.80 (scheme shown on left). The systems shown in **a** and **b** each consist of a pair of nanoparticles; one has an Au core diameter of 15 nm and the other is 5 nm. In the case of the small particle, the hydrodynamic radius was deliberately changed by changing the thickness of the DNA shell so that the *β*/*α* in **b** is greater than in **a**. The system in **c** used the same 15 nm Au core PAEs that were used in **a** and **b**, but the hydrodynamic radius was varied by changing the Au core diameter of the small particle (10 nm in diameter) so that the *β*/*α* in **c** is greater than in **a**. In the SAXS patterns, the black trace is the experimentally obtained scattering pattern, and the red trace is the predicted scattering pattern for a model lattice. The lattice parameters from SAXS results were used to calculate the *c*/*a* ratio of each unit cell. Scale bars are 1 μm

Faceted hexagonal prism microcrystals were obtained with ten different unit cells that are isostructural with the AlB_2_ lattice, but with different *a* and *c* values (Fig. 2, Supplementary Fig. 8). The AB_2_ colloidal crystal superlattice (space group 191) is a hexagonal unit cell with two distinct types of particles, where the structure consists of layers of hexagonally arranged large particles with small particles in every trigonal prismatic interstice (Fig. 1b bottom). Unlike the AlB_2_ structure found in atomic systems where the *c/a* ratio is fixed to 1.08, the *c/a* ratio for PAE superlattices is tunable and controlled by the hydrodynamic radius of each component, which can be varied by changing the nanoparticle size, DNA length, or both (Fig. 2). The rationale for achieving a range of *c/a* ratios with identical crystallographic symmetries can be described using a basic principle in colloidal crystal engineering with DNA: colloidal crystals generated from rigid nanoparticles and soft, DNA ligand shells can tolerate unfavorable repulsive interactions if the system can access a more thermodynamically stable structure (i.e., the PAEs can elastically compress along the *c*-axis or across the *ab*-plane if they can be in more thermodynamically favorable positions)^30–32^. Importantly, the fact that the *c/a* ratio is tunable suggests that the relative strength of attractive and repulsive forces between PAEs can be varied using individual PAE structure as a design parameter. Furthermore, we show that salt concentration can alter the *c/a* ratio (Supplementary Fig. 8). The DNA shells around the nanoparticle core can be compressed with the addition of salt, which decreases the repulsion between DNA strands within the overlap region and results in compression of the superlattice. Significantly, this is not isotropic since the lattice itself is not symmetric (Supplementary Fig. 7). With these observations, one can choose PAEs and salt conditions to produce microcrystals with structural parameters that favor the Wulff shape (Supplementary Fig. 9).

## Discussion

**Fig. 3:**
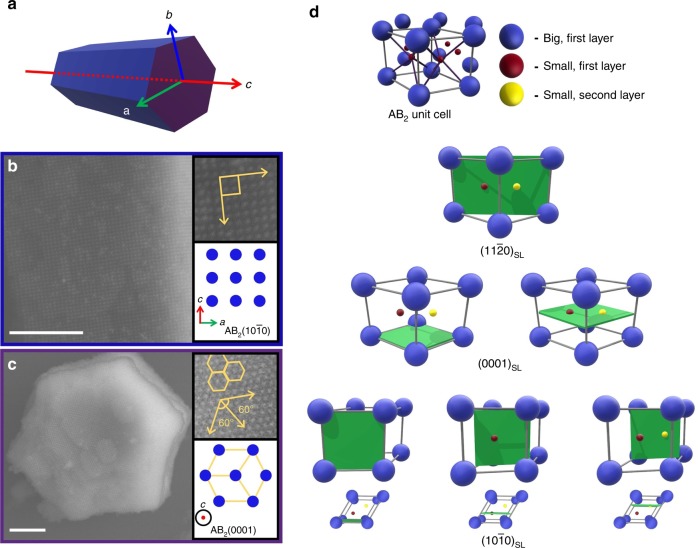
Surface features of hexagonal prism microcrystals. **a** Scheme of a hexagonal prism and **b**–**c** SEM images of **b** a rectangular face with a (10$\overline 1$0)_SL_ (inset) and **c** a hexagonal base with a (0001)_SL_ (inset) of a hexagonal prism. For clarity, rectangular faces and a hexagonal base are color coded as blue and purple both in the scheme and SEM images, respectively. Scale bars are 500 nm. **d** Scheme of an AB_2_ unit cell and the projections of the (11$\overline 2$0)_SL_, (0001)_SL_, and (10$\overline 1$0)_SL_ planes. The plane multiplicity can be determined by counting the number of different planes (green slabs) that correspond to the same Miller indices. Nanoparticles in different colors may occupy different layers of a plane with identical Miller indices. Gray and purple lines in the model lattice denote the edges of the unit cell and the bonds that connect complementary particles, respectively. Top–down views of different planes of the (10$\overline 1$0)_SL_ facet are shown for clarity

To fully understand why such crystals form, one needs to understand the growth kinetics of such structures. Indeed, the formation of hexagonal prisms from an AB_2_ packing of nanoparticles can be explained by the kinetic growth of the exposed facets, where the crystal is enclosed by the slowest-growing facets. In other words, kinetic anisotropy in the growth rates can dictate shape selection, as described by the kinetic Wulff construction^33^. Wulff made a direct correlation between the surface energy and growth rate^1^. This assumption led to the conclusion that the lowest surface energy facet, which has the slowest growth rate in simple systems, is exposed in the final shape. Previously observed Wulff polyhedra formed via colloidal crystal engineering with DNA were formed from one-component systems^28,29^, where all particles are equivalent from a kinetic standpoint, and thus the surface energy can be directly correlated to the growth rate to predict the thermodynamic shape. This correlation does not hold in all cases, specifically in multicomponent crystallization. In a binary system such as an AB_2_ lattice, the diffusion and attachment kinetics of each component (A and B) are expected to be different, and this can significantly contribute to the observed crystal shape, resulting from either diffusion-limited or interface-limited growth^33–35^. Furthermore, in contrast with one-component cubic lattices, the same Miller indices can be assigned to different planes. Indeed, unlike previously observed Wulff polyhedra^28^, the lowest surface energy facet is not exposed in these AB_2_ crystals (Supplementary Fig. 3). Based on the calculation of relative effective surface energies (γ_eff_) for different AB_2_ facets (Supplementary Table 5, Supplementary Fig. 1), the AB_2_(11$\overline 2$0) plane has the lowest surface energy. The equilibrium crystal structure predicted from a Wulff construction is not the experimentally observed elongated hexagonal prism morphology, confirming that the observed hexagonal prismatic crystals are non-equilibrium products (Supplementary Fig. 3, 4). Consistent with this conclusion, SEM analysis of the surface a microcrystal reveals that the hexagonal prisms are enclosed by the highest surface energy facets: the rectangular faces are (10$\overline 1$0) facets (Fig. 3a, b, d) and the hexagonal ends are (0001) facets (the facet normal to the (10$\overline 1$0) facet; Fig. 3a, c, d).

**Fig. 4:**
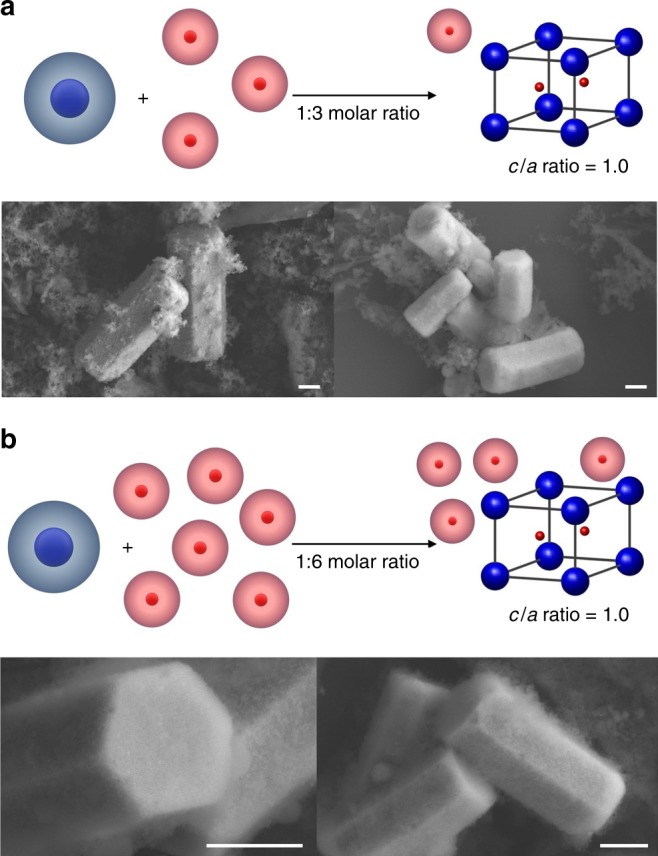
Hexagonal prisms produced upon addition of off-stoichiometric ratios of PAEs. **a**, **b** Scheme and SEM images of hexagonal prism microcrystals when different molar ratios of A:B particles, (**a**) 1:3 and **b** 1:6, were added. In both cases, AB_2_ lattices have a similar *c*/*a* ratio to the one formed using a stoichiometric ratio of PAEs (1:2). Scale bars are 1 μm

The crystal growth rate is dependent upon many variables (e.g., PAE diffusion rate, chemical potential, termination effect) that vary as a function of both particle and facet type^33,34^. For the PAE system where the crystals are grown by slow cooling over several days through the melting temperature (i.e., particles were given enough time to equilibrate), we hypothesize that single-crystal formation is less likely to be diffusion-limited. To explore whether the difference between the chemical potentials of each component plays a role in crystal growth, off-stoichiometry (i.e., a ratio of A:B particle mixtures not equivalent to 1:2) experiments were carried out with 1:3 and 1:6 molar ratios of A:B. The observed crystal habits in these systems are the same as those observed in the stoichiometric system, suggesting that the chemical potential difference has a negligible influence on crystal shape (Fig. 4). For multicomponent systems, the composition of the particle (type) terminating the crystal surface can affect the overall growth kinetics, as described by termination effects observed in atomic crystals, for example, III–V semiconductors such as GaAs^36^. For an AB_2_ lattice, each crystal plane corresponding to a set of identical Miller indices can exhibit a multiplicity up to three; that is, the number of distinct planes with the same Miller indices is three (Fig. 3d). For example, the AB_2_(10$\overline 1$0) plane has a multiplicity of three (blue, red, or yellow) while the AB_2_(11$\overline 2$0) plane has a multiplicity of one as it intersects all particles within a unit cell (Fig. 3d, Supplementary Fig. 2). We therefore calculated the actual surface energies for different surface terminations using MD simulations. These calculations reveal that different surface terminations have different surface energies when a set of Miller indices exhibits a multiplicity greater than one, and this difference in surface energy, which is the largest for the AB_2_(10$\overline 1$0) plane, creates a nucleation barrier for the growth of the subsequent layer (Table 1). This is especially true when a layer with a higher surface energy nucleates on top of a layer with a lower energy.

**Table 1:** Relative difference in surface energy values between different layers calculated for DNA-gold nanoparticle superlattices

Layer Type	Δγ_plane_(11$\overline 2$0)	Δγ_plane_(0001)	Δγ_plane_(10$\overline 1$0)	Δγ_plane_(11$\overline 2$1)	Δγ_plane_(10$\overline 1$1)
1	—	0.093	−0.079	−0.030	0.022
2	—	−0.093	−0.122	0.030	−0.142
3	—	—	0.201	—	0.120

Δ*γ*_plane_ represents difference in surface energy values (in kJ mol^−1^ nm^−2^) for different layers of a set of identical Miller indices

**Fig. 5:**
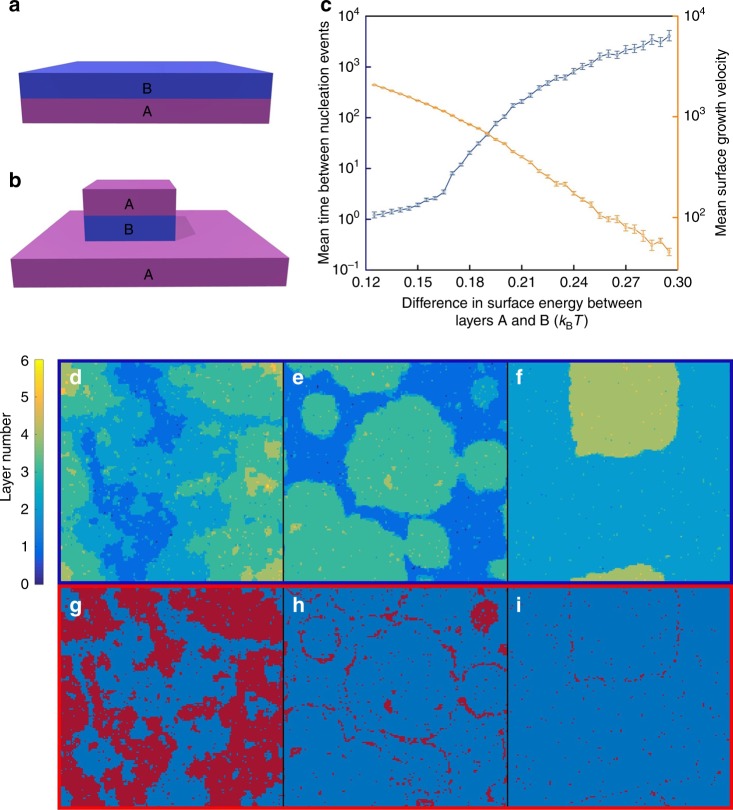
Kinetics of epitaxial growth of alternating layers of A and B calculated using Monte-Carlo simulations (*γ*_edge_ = 0.65 *k*_B_*T*, Δ*µ* = −0.3 *k*_B_*T*). **a**, **b** Representative scheme of layer growth mechanisms in the presence of a minimal or no energy barrier (**a**) and a substantial energy barrier between A and B layers (**b**). **c** The mean time between nucleation events and the mean surface growth velocity plotted as a function of the energy barrier between layer growth. Error bars are from standard deviation and indicate 95% confidence interval. **d**–**f** Typical surface profiles for Δ*γ*_A-B_ = 0 *k*_B_*T*, 0.12 *k*_B_*T*, and 0.29 *k*_B_*T*, respectively. **g**–**i** The same surface profiles colored with the low energy layer in blue and the high energy layer in red. The color scheme presented in **g**–**i** allows one to clearly view the placement of the high energy layer. As the energy barrier increases between layers, the presence of layer B is suppressed and is eventually only found near the terrace edges

For the typical kinetic Wulff shape construction, the growth of layers occurs through the formation of a terrace, which then forms a complete monolayer before the subsequent growth of the next layer (Fig. 5a). For such growth, the growth rate, *r*, can be expressed as *r* = *f*_0_e^(−*E*/*k*B*T*)^, where *f*_0_ is the prefactor and E is the nucleation barrier (*E* = −π(γ_edge_)^2^/Δ*µ*; γ_edge_ is the edge energy per atom of a terrace and Δ*µ* is the bulk chemical potential drive, which is negative for crystal growth)^33^. For facets with a multiplicity greater than one, the nucleation of an additional particle results in a change in the overall surface energy akin to a change in the bulk chemical potential (Table 1). When the growth of a layer is energetically unfavorable, the nucleation of a subsequent layer may occur before growth of the initial layer is complete, resulting in multi-terrace type growth (Fig. 5b). To better understand the growth mechanism relevant to our system, we derived equations for a simple two-layer system, where layer “A” has lower surface energy than layer “B.” The energy barrier *E* for the growth of A on B and B on A are given by *E*_A/B_ = −π(γ_edge_)^2^/(Δ*µ* + Δ*γ*_A-B_) and *E*_B/A_ = −π(γ_edge_)^2^/(Δ*µ* − Δ*γ*_A-B_), respectively, where Δ*γ*_A-B_ is the difference in surface energy between layers A and B (measured per particle). Based on this model, when |Δ*µ*| is same as |Δ*γ*_A-B_|, there is no thermodynamic driving force for the nucleation of the higher surface energy layer B. In this case, the crystal growth proceeds through the growth of a BA bilayer as the growth of A on B occurs significantly faster than the growth of B on A. Therefore, E_BA_ can be written as −2π(γ_edge_)^2^/Δ*µ*. If this is the growth mechanism that the PAEs undergo, one would expect to recover the thermodynamic Wulff shape, however, this is not experimentally observed (Supplementary Fig. 3). Therefore, we hypothesize that the growth mechanism that leads to the formation of hexagonal prism microcrystals from an AB_2_ packing of nanoparticles is layer-by-layer growth, as described in the kinetic Wulff construction.

To investigate this hypothesis, we used a solid-on-solid model in kinetic Monte-Carlo simulations to evaluate the effect of a nucleation barrier on the growth kinetics for the plane with a multiplicity of two. Here, layer growth can proceed through two different pathways as illustrated in Fig. 5d–i (Supplementary Fig. 5, 6, see Kinetic Monte-Carlo Simulations section of Supplementary Information). At lower values of Δ*γ*_A-B_ (below 0.24 *k*_B_*T* in Fig. 5c), layer growth is significantly slowed but still proceeds in a layer-by-layer fashion. Whereas, when the Δ*γ*_A-B_ value is above ~0.24 *k*_B_*T* (where the curve starts to plateau), we observe the formation of BA terraces (Fig. 5b, f, i). Thus, for the microcrystals experimentally observed, the PAEs are expected to exhibit a layer-by-layer growth behavior, which results in a non-equilibrium Wulff crystal (Fig. 5d). The strong parallels between the energy barrier calculations and simulation results indicate that the plane with the largest Δ*γ*_A-B_ is present in the final shape through a layer-by-layer growth mechanism. Importantly, the kinetic Wulff shape, a hexagonal prismatic crystal bounded by the (10$\overline 1$0) and (0001) facets, is predicted by setting the growth speed of the AB_2_(10$\overline 1$0) (the facet with the greatest Δ*γ*_A-B_ in our system) to zero (Supplementary Fig. 4).

This work explains how one can use DNA to engineer non-equilibrium colloidal single crystals with uniform anisotropic shapes. Although we have focused on one specific system through a detailed study, the plane multiplicity mechanism introduced here is general and, in principle, can be used to access a variety of non-equilibrium Wulff shapes, thereby significantly broadening the scope of structures possible through colloidal crystal engineering with DNA.

## Electronic supplementary material

Supplementary Information

## Data Availability

The authors declare that the main data supporting the findings of this study are available within the article and its Supplementary Information files. MD simulations were performed with the HOOMD-blue software package^37,38^, with initial configuration built with the HooBAS package (https://bitbucket.org/NUAztec/hoobas). Kinetic Monte-Carlo simulations were performed using an in-house code available at https://bitbucket.org/NUAztec/epitaxial-kmc. Raw data for simulations are available through the open science framework, 10.17605/OSF.IO/9QYZA. The authors declare that all relevant data are available from the authors upon reasonable request.
