# Supplementary material for: Non-equilibrium anisotropic colloidal single crystal growth with DNA
Source: Nat Commun. 2018 Nov 1;9:4558. doi: 10.1038/s41467-018-06982-9 (PMC6212572; doi:10.1038/s41467-018-06982-9)
Supplement: Supplementary file 1 — Supplementary Information [file 41467_2018_6982_MOESM1_ESM.pdf]

Supplementary Information for

Non-Equilibrium Anisotropic Colloidal Single Crystal Growth With DNA

Soyoung E. Seo,[1,2] Martin Girard,[3,4] Monica Olvera de la Cruz,[1,2,3,4] and Chad A. Mirkin[1,2,3*]*

[1]Department of Chemistry, [2]International Institute for Nanotechnology, [3]Department of Materials Science and Engineering, [4]Department of Physics and Astronomy, Northwestern University, Evanston, Illinois 60208, United States

*Address correspondence to: m-olvera@northwestern.edu and chadnano@northwestern.edu

Supplementary Methods

Synthesis and Assembly of DNA-Nanoparticle Conjugates

All oligonucleotides were synthesized on a MM48 solid-support automated DNA synthesizer (BioAutomation) using standard phosphoramidite chemistry (Supplementary Table 1). All reagents were purchased from Glen Research (Sterling, VA). DNA strands were purified by reverse phase HPLC (Varian RP-HPLC) on an Agilent C18 column, followed by standard deprotection protocols[1]; the acid-labile 4,4'-dimethoxytrityl (DMT) protecting group was cleaved off the DNA by incubating in a mL of 20% acetic acid for 1 hour. All oligonucleotides were characterized by matrix-assisted laser desorption/ionization time-of-flight (MALDI-TOF) mass spectroscopy. DNA concentration was measured on a Cary 5000 UV-Vis-NIR spectrophotometer (Agilent) using extinction coefficients from the IDT OligoAnalyzer (https://www.idtdna. com/calc/analyzer).

For DNA-functionalization of gold nanoparticles (AuNPs), 5, 10, and 15 nm diameter citrate-capped AuNPs (Ted Pella) were used as received with no further purification. DNA functionalization of AuNPs was carried out with propyl thiol-modified oligonucleotides (Sequences **1-3**, Supplementary Table 1). Thiolated oligonucleotides were treated with a solution of 100 mM dithiothreitol (DTT) for 1 h, which was then purified using Nap-5 size exclusion column (GE Healthcare) to remove remaining DTT. Deprotected oligonucleotides were immediately added to a AuNP solution in a ratio of approximately 6 nmol of DNA per 1 mL of Au colloid, followed by addition of the surfactant sodium dodecyl sulfate (SDS) to bring the final surfactant concentration to 0.01 *vol*%. Initial addition of the purified thiol-modified DNA allows the formation a low-density monolayer of oligonucleotides on the nanoparticle's surface. The DNA loading was increased using a "salt aging" process[2], where 5 M NaCl solution was slowly added over the course of a few hours to bring the final NaCl concentration to 0.5 M. In between each addition of salt, the solution was vortexed and sonicated for a few seconds. After bringing the final NaCl concentration to 0.5 M, the AuNPs were incubated in a shaker overnight at 37°C at 130 rpm. Unbound DNA, excess salt, and surfactant were removed by three rounds of centrifugation in 100 kDa membrane filter centrifuge tubes (Millipore) on a swinging bucket centrifuge (2200 rpm) and suspension in Nanopure water (18.2 MΩ, Millipore). DNA-functionalized nanoparticle concentration was quantified on a Cary 5000 UV-Vis-NIR spectrophotometer using known extinction coefficients from the Ted Pella website (http://www.tedpella.com/gold_html/gold-tec.htm).

DNA-AuNPs were assembled into superlattices through the addition of complementary DNA linker strands (Sequences **4-7**). The linker strands contain a "duplexer region" that is complementary to the sequence attached to the AuNPs and a "sticky end region" that is a short six ($^{5'}$TTCCTT$^{3'}$ and $^{5'}$AAGGAA$^{3'}$) to seven base ($^{5'}$TTCCTTT$^{3'}$ and $^{5'}$AAAGGAA$^{3'}$) pair-long sequence that engages in hybridization to the complementary sticky end on neighboring AuNPs. A single base "flexor" is located between the sticky end and the duplexer region to provide flexibility to the sticky end during nanoparticle assembly. Different numbers of hexaethylene glycols (sp18s) were incorporated into the thiol-modified DNA located near the nanoparticle surface to provide flexibility to the overall strand and control the DNA shell thickness. For 5, 10, and 15 nm AuNPs, the DNA linker strands were added in a molar ratio of 1:150, 1:250, and 1:400, respectively, to ensure maximal linker loading. Then, an appropriate amount of 5 M NaCl was added to these solutions to reach the final NaCl concentrations of either 1 or 2 M. Upon addition of the DNA linker strands and salt, the DNA-AuNP solution changed from red to purple, indicative of a plasmonic red shift and aggregate formation. Visible aggregates were formed and transferred to 200 µL PCR tubes (Applied

Biosystems) for slow cooling. Prior to thermal annealing, the melting temperature was measured by monitoring the extinction of the AuNPs at 520 nm while increasing the temperature from 25°C to 65°C at a ramp rate of 0.1°C/10 min using UV-Vis spectrophotometer. The melting temperature was determined by taking the derivative of the melting curves and finding the x-value corresponding to the maxima.

Supplementary Table 1. DNA Sequences.

**Thiol-Modified Strands**		
**#**	**Sequence Name**	**Sequence (5' to 3')**
**1**	Nanoparticle-bound Strand	HS-$(sp18)_2$-CAT CCA TCC TTA TCA ACT
**2**	Nanoparticle-bound Strand	HS-$(sp18)_5$-CAT CCA TCC TTA TCA ACT
**3**	Nanoparticle-bound Strand	HS-$(sp18)_5$-AAC GAC TCA TAC TCA CCT
**Linker Strands**		
**#**	**Sequence Name**	**Sequence (5' to 3')**
**4**	Linker for **1** and **2**	TT CC TT-(sp18)-AGT TGA TAA GGA TGG ATG
**5**	Linker for **3** (pair with **4**)	AA GG AA-(sp18)-AGG TGA GTA TGA GTC GTT
**6**	Linker for **1** and **2**	TT CC TTT-(sp18)-AGT TGA TAA GGA TGG ATG
**7**	Linker for **3** (pair with **6**)	AAA GG AA-(sp18)-AGG TGA GTA TGA GTC GTT

*One sp18 is equivalent to one hexaethylene glycol unit manufactured by Glen Research.

Hydrodynamic Radius Calculation

The hydrodynamic radius of a PAE refers to the combination of the inorganic nanoparticle radius and the length of the DNA. To successfully synthesize AB_2 lattices (isostructural with aluminum diboride), the hydrodynamic radius of a PAE was estimated for each particle design. The hydrodynamic radius calculation of a DNA linker loaded PAE assumes DNA duplexes are in canonical B-form, fully outstretched and perpendicular to the nanoparticle's surface. The length of a sp18 monomer was assumed to be 1.0 nm based on previous work[3]. Based on these assumptions and experimentally determined values, the hydrodynamic radius was approximated as:

$$R_h = R_{NP} + 0.34n_{bp} + 0.8n_{sp18\ NP\ bound} + 0.2n_{sp18\ Linker} + 0.4 \qquad \text{Supplementary Eq. 1}$$

where R_h is the hydrodynamic radius of a PAE, R_{NP} is the radius of an inorganic nanoparticle core, n_{bp} is the number of DNA bases, and n_{sp18} is the number of sp18s. The calculated values were used to calculate the approximate particle size ratios for the superlattice design. For example, if the size ratio between two PAEs was equivalent to 1, the resulting superlattices will have a body-centered cubic crystallographic symmetry, instead of AB_2 crystallographic symmetry[4].

Single Crystal Growth by Slow Cooling

The final concentration of nanoparticles was 75 nM in a total volume of 100 µL. The transition from amorphous aggregates to crystalline superlattices was initiated by slowly cooling from a temperature above the melting temperature to room temperature in a PCR Thermocycler (Life Technologies) at a cooling rate of 0.1°C/10 min. As the PAEs were heated above their melting temperature, the aggregates completely dissociated. As the PAEs were cooled below the melting temperature, they begin to assemble to form

micron-scale single crystals. The starting temperature was chosen based on the sticky end strength, which changes with sequence, linker loading density, and salt concentration, and either 58°C, 55°C, or 50°C was chosen depending on the samples being slow cooled. For example, a seven base-long sticky end exhibits an enhanced bond strength, and therefore a higher starting temperature was chosen then for a six base-long sticky end sample. The full slow cooling procedure took ~2-3 days.

Small Angle X-ray Scattering

All SAXS experiments were performed at the DuPont-Northwestern-Dow Collaborative Access Team (DND-CAT) beamline 5ID-D at the Advanced Photon Source (APS) at Argonne National Laboratory. The data were collected with 10 keV (wavelength 1.24 Å) collimated X-rays calibrated against a silver behenate standard. The X-ray beam cross-section of 200 μm and a 0.5 s exposure time were used. Longer periods of X-ray exposure damaged the crystals. Approximately 60 μL of the sample was loaded into a 1.5 mm quartz capillary (Charles Supper) and placed into a sample stage in the path of the X-ray beam. Dark current frames were subtracted from all data. Two-dimensional scattering data were collected on a CCD area detector and converted to one-dimensional data *via* radial averaging to generate plots of scattering intensity $I(q)$ as a function of the scattering vector q ($q = 4\pi \sin(\theta)/\lambda$, where θ is the scattering angle and λ is the wavelength of the X-ray radiation used). To index the resulting scattering patterns, the experimental data was compared with patterns modeled from ideal superlattice structures of AlB_2 lattice symmetry generated in MATLAB. More detailed description about the peak fitting procedure can be found in reference 5.

Lattice Parameter Calculation from the SAXS Data

Supplementary Table 2. Constants for Lattice Parameter Calculations.

Lattice Type (Space Group)	q_0[hkl]	Nearest Neighbor Position	C	Lattice Parameter *a* (Å)	Lattice Parameter *c* (Å)
AlB_2 (191)	[001]	[1/3,2/3,1/2]	2π	$C/q_{[100]}$	$C/q_{[001]}$

All relevant values were calculated using the SAXS data collected for a solution-phase sample. The lattice parameters are the edge lengths of the unit cell, given by *a*, *b*, and *c*. For the AlB_2 lattice, $a = b \neq c$ (Fig. 1b). Therefore, for the AB_2 structures, multiple *c/a* ratios (including 1 in the case $a = c$) are observed depending on the lattice design and thus the lattice parameters *a* and *c* were calculated independently (Supplementary Table 2). A value of C is a constant that correlates the distance between two nearest neighbors and the distance between the [*hkl*] planes associated with the scattering peak and is used to calculate the lattice parameters. The nearest neighbor distance d (in nm) between particles A and B in a superlattice can be extrapolated as:

$$d = \frac{1}{10}\left(\left(\frac{c}{2}\right)^2 + \left(\frac{\sqrt{3}a}{3}\right)^2\right)^{-1/2}$$ Supplementary Eq. 2

where *c* is the lattice parameter in the *z*-direction and *a* is the lattice parameter in the *x*-direction (in Å). It is important to note that the X-ray beam signals are predominantly from the scattering by the inorganic

nanoparticle cores (excluding the oligonucleotides). Using these calculated values, the relative positions of different types of particles can be determined within the lattice.

Scanning Electron Microscopy (SEM)

Slow-cooled solution-phase crystals were transitioned to the solid state *via* silica encapsulation using literature methods[3]. Embedding in the solid-state is necessary for visualization by electron microscopy because the structural parameters will change if the lattices are prepared for imaging by drying. Slow-cooled samples were transferred to a 1.5 mL Eppendorf tube, and the volume was brought up to 1 mL with a solution at an appropriate NaCl concentration. To this solution, 2 µL of the quaternary silane salt, *N*-trimethoxysilylpropyl-*N,N,N*-trimethylammonium chloride (TMSPA), was added and the tube was mixed at room temperature on a thermomixer (Eppendorf) at a rate of 700 rpm. After the TMSPA was allowed to electrostatically associate with the negatively charged DNA backbone for about 30 min, 4 µL of triethoxysilane (TES) was added to the solution and allowed to form a silica network around the entire lattice. The mixture was left on the thermomixer at 700 rpm for four days, followed by three rounds of centrifugation, removal of supernatant, and resuspension in Nanopure water. Note that the addition of water does not result in lattice dissolution (*i.e.,* the solution turns red) in the case of successful silica embedding. It is often impossible to remove excess silica chunks, which are often appeared, during the purification process.

SEM images were taken at the Northwestern University Atomic and Nanoscale Characterization Experimental Center (NUANCE) on a Hitachi SEM SU8030 instrument at an accelerating voltage of 10 kV using either SE_L or BSE_L detectors. Silica embedded crystals were resuspended in approximately 100 µL of 50% ethanol. Approximately 5 µL of the solution was dropcast onto a silicon wafer for imaging.

Molecular Dynamics (MD) Simulations

To explore the contribution of surface energies of different facets on the shape selection, molecular dynamics simulations were carried out to understand the origin of facet yield changes. The coarse-grained approach used in this work is the same as the one demonstrated in previous literature[4], which used a model first developed by Knorowski *et al*[6] and then adapted for double-stranded DNA (dsDNA) by Li *et al*[7]. MD simulations used a PAE design similar to the one used in experiments.

To calculate the surface energies of a unit cell, we used two different systems, a perfect 3D crystal and a slab of finite thickness in the *z*-direction, with a defined surface plane (Supplementary Fig. 1). Distances between the nanoparticles were determined from the equilibrated lattices. To equilibrate a lattice, we initialized colloids in a perfect crystal and let the system relax using the isobaric-isothermal ensemble (NpT). We equilibrated systems of $4 \times 4 \times 4$ and $6 \times 6 \times 6$ unit cells with relative differences in mean energy and lattice parameters of less than 10^{-3}. We considered a few, different systems with varying DNA lengths to find the system similar to the experimental one, while keeping the particle sizes constant. The double-stranded portion was fixed for all systems while the single-stranded segment (ssDNA), which represents the PEG linker region in our DNA design, was varied. Supplementary Table 3 summarizes the values used for lattice equilibration.

Supplementary Table 3. DNA Lengths and Equilibrated Lattices.

System	dsDNA Length (# of bases)	Particle A ssDNA Length (# of bases)	Particle B ssDNA Length (# of bases)	*a* (nm)	*c* (nm)	*c/a*
A	16	10	4	40.52	36.51	0.901
B	20	10	4	44.34	39.56	0.892
C	16	10	10	42.31	37.00	0.875
D	16	14	14	44.50	38.82	0.872

*Core sizes of particles A and B were 5 nm and 15 nm, respectively.

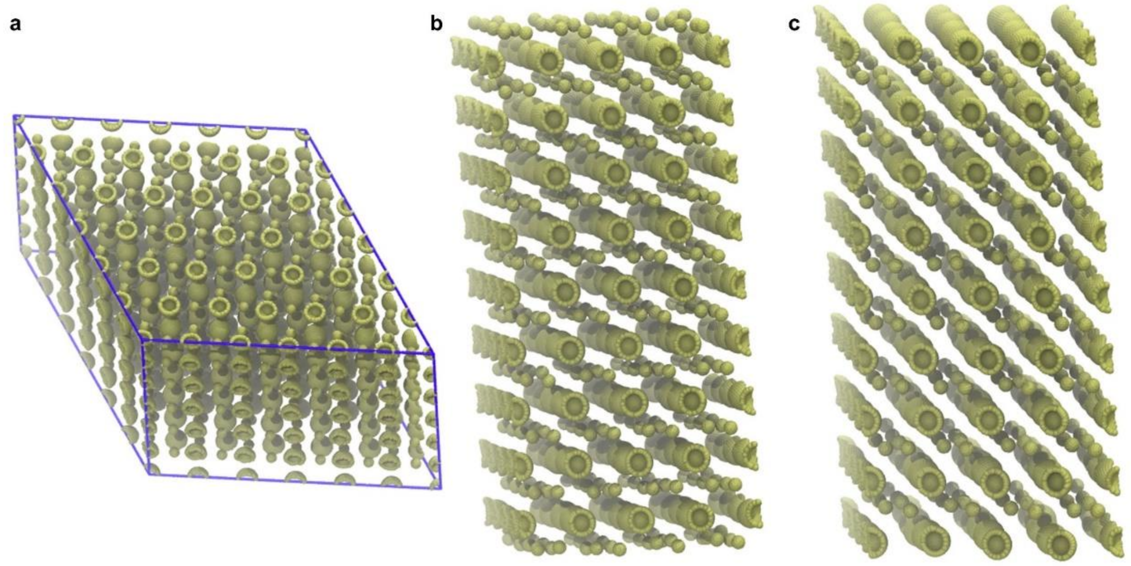

Supplementary Figure 1. Simulation snapshots of **a,** a perfect 3D crystal with 6 × 6 × 6 unit cells, and **b** and **c,** surface slabs showing two different terminations of the (0001) plane.

As for our previous surface energy calculations[8], we did not fix the positions of the colloids. This allows the surfaces to undergo simple reconstructions where the colloids are displaced with respect to the bulk crystal lattice. Each crystal slab has at least six crystal periods in the *z*-direction and 4 periods in the *x*- and *y*-directions. Crystal slabs typically have 112 to 160 colloids of type A and 256 to 320 colloids of type B, yielding a typical 650k to 1M beads in the system. Since the unit cell of an AB_2 crystal has three distinct points, we have up to three different surface terminations. The three different terminations were evaluated for all Miller indices, including the ones that are equivalent to confirm that the surface energies calculated herein are reproducible. Calculations were performed twice for each set of Miller indices. Tools to generate initial configurations can be found online (https://bitbucket.org/NUaztec/hoobas). The number of distinct surface terminations for a given set of Miller indices depends on the number of points crossed in the unit cell (Fig. 3d, Supplementary Fig. 2). Multiplicity numbers for different planes are given in Supplementary Table 4.

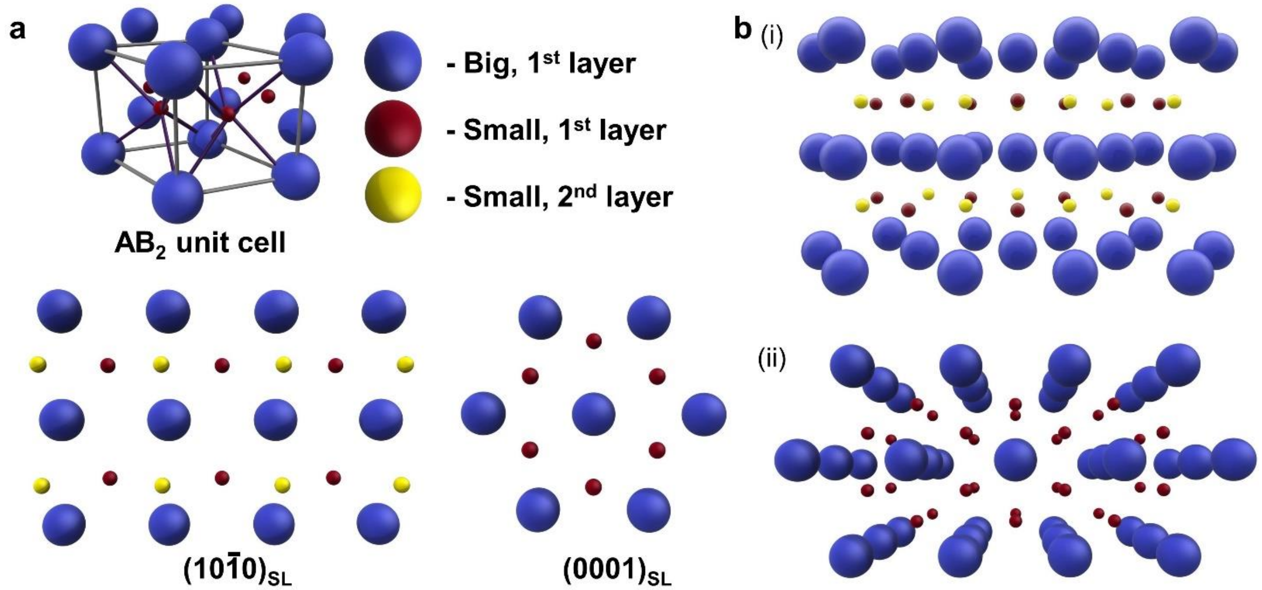

Supplementary Figure 2. a, Scheme of an AB_2 unit cell and the projections of $(10\bar{1}0)_{SL}$ and $(0001)_{SL}$ planes. Nanoparticles in different colors may occupy different layers of a plane with the identical Miller indices. **b,** Scheme of extended projections of (**i**) $(10\bar{1}0)_{SL}$ and (**ii**) $(0001)_{SL}$ planes for a better visualization.

Supplementary Table 4. Summary of Multiplicity of Different Set of Miller Indices.

Plane	$(11\bar{2}0)$	(0001)	$(10\bar{1}0)$	$(11\bar{2}1)$	$(10\bar{1}1)$
Multiplicity	1	2	3	2	3

The model uses beads which interact through Lennard-Jones (LJ) interactions, harmonic bonds and harmonic angle interactions. The bead diameters are per bead type to be ssDNA = 1 nm, dsDNA = 2 nm, DNA backbone = 1 nm, flanking beads = 0.6 nm, sticky beads = 0.6 nm and surfaces = 2 nm. The LJ pair interaction for two beads of type A and B located at distance r is described by:

$$U_{\mathrm{LJ}} = \epsilon_{\mathrm{AB}}\left(\left(\frac{\sigma}{r}\right)^{12} - \left(\frac{\sigma}{r}\right)^{6}\right) \quad \text{Supplementary Eq. 3}$$

where r_c is the cutoff after which the energy is set to zero and $U_{\mathrm{LJ}}(r_c)$ is the energy at the cutoff. All repulsive interactions have $\varepsilon = 1$ and $r_c = \sigma\, 2^{1/6}$ (Weeks-Chandlers-Andersen potential). Only complementary bases are attractive with $\varepsilon = 7$ and $\sigma = 2$. All $\sigma_{AB} = (d_A + d_B)/2$ with exception of the following pairs: DNA backbone–sticky end: 1.2 nm, flanking bead–sticky end: 0.86 nm, dsDNA–sticky end: 0.91 nm, flanking bead–flanking bead: 0.8 nm, sticky end–sticky end: 2 nm, surface-surface: 10^{-5} nm. The harmonic bond potential is defined by $U_B = 1/2\, k_B\, (r - r_0)^2$, with the dsDNA having $k_B = 82.5$ nm^{-2} and $r_0 = 1.68$ nm. Parameters for harmonic bond and angle potentials can be found in the following work[5].

The surface energy of a given crystal plane was obtained using the difference in enthalpy between the crystal slab with exposed facets (Supplementary Fig. 1) and a perfect crystal. The surface enthalpy is given by:

$$\gamma_{plane} = \frac{U_p - U_A n_A - U_B n_B}{A_p} \quad \text{Supplementary Eq. 4}$$

where γ_{plane} is the surface energy of a given plane, U_{p} is the enthalpy of the crystal slab with exposed plane p, U_{A} (or U_{B}) is the mean energy of colloids of type A (or B) in a perfect crystal, and n_{A} (or n_{B}) is the number of colloids of type A (or B) in the crystal slab. The mean of the relative variation between repeat calculations of the surface enthalpy is around 4%.

Simulations are performed in the NVT ensemble using a Langevin thermostat (friction coefficients of 1.0). The crystal structure is thermally equilibrated over 1.5×10^6 timesteps, while energy is measured with 500 values taken over 2.5×10^6 timesteps with a timestep of $\Delta t = 3 \times 10^{-3}\ \delta t$ and $\delta t = (\mathrm{m}\ \sigma^2/\varepsilon)^{1/2}$.

Supplementary Table 5. Effective Surface Energy Values Calculated AB_2 Superlattice.

Name	$\gamma_{eff}(11\bar{2}0)$	$\gamma_{eff}(0001)$	$\gamma_{eff}(10\bar{1}0)$	$\gamma_{eff}(11\bar{2}1)$	$\gamma_{eff}(10\bar{1}1)$	Ratio of Surface Energies
A	0.508	0.721	0.529	0.534	0.510	1:1.42:1.04:1.05:1
B	0.411	0.571	0.431	0.424	0.404	1:1.39:1.05:1.03:0.98
C	0.474	0.649	0.485	0.487	0.47	1:1.37:1.02:1.03:0.99
D	0.425	0.590	0.440	0.445	0.434	1:1.39:1.04:1.05:1.02

*γ_{eff} represents effective surface energy (in kJ mol^{-1} nm^{-2}), which is the lowest surface energy value among values calculated for different terminations.

Supplementary Table 6. Slab Calculation Values for Different Terminations.

Name	Termination Type	$\gamma_{plane}(11\bar{2}0)$	$\gamma_{plane}(0001)$	$\gamma_{plane}(10\bar{1}0)$	$\gamma_{plane}(11\bar{2}1)$	$\gamma_{plane}(10\bar{1}1)$
A	1	0.508	0.814	0.651	0.534	0.652
A	2		0.721	0.529	0.564	0.510
A	3			0.730		0.630
B	1	0.411	0.614	0.546	0.424	0.528
B	2		0.571	0.431	0.451	0.404
B	3			0.576		0.495
C	1	0.474	0.717	0.612	0.487	0.608
C	2		0.649	0.485	0.539	0.470
C	3			0.685		0.569
D	1	0.425	0.662	0.573	0.445	0.547
D	2		0.590	0.440	0.472	0.434
D	3			0.597		0.518

*γ_{plane} represents surface energy values (in kJ mol^{-1} nm^{-2}) for different terminations of a plane with identical Miller indices. Termination type 1 always has the large particles on the surface.

Wulff Shape Identification

The thermodynamic equilibrium shape of a lattice is governed by the stability of crystal surfaces, and thus the shape can be computationally predicted based on surface energy values. Using surface energy values estimated from the MD simulations, we calculated the ratio of surface energies and used the WulffMaker software to predict corresponding Wulff polyhedra[9]. Supplementary Fig. 3 shows the corresponding thermodynamic Wulff polyhedra predicted from the surface energies for the lattice with experimentally determined lattice parameters.

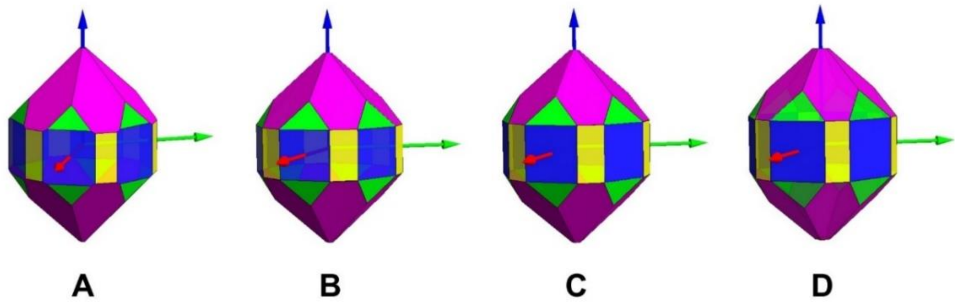

Supplementary Figure 3. A-D, Wulff polyhedra simulated for different systems (see Supplementary Table 3 for details about systems A-D) exhibit similar crystal habits and deviate from the experimental results. All crystals are truncated by the $AB_2(11\bar{2}0)$ blue, $AB_2(0001)$ purple, $AB_2(10\bar{1}0)$ yellow, $AB_2(11\bar{2}1)$ green, and $AB_2(10\bar{1}1)$ magenta facets.

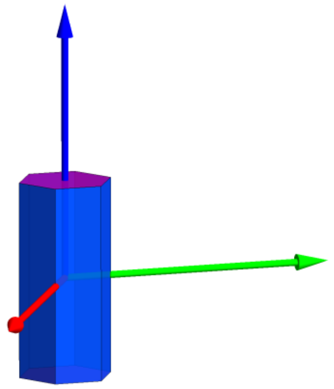

Supplementary Figure 4. Wulff equilibrium shape is predicted for the case when the growth velocity of the $AB_2(10\bar{1}0)$ is assumed to be very slow. Because the Wulffmaker software can only generate the shape based on the surface energy values, the surface energy of the $AB_2(10\bar{1}0)$ was set as the lowest value, which is equivalent to the slowest growth velocity). Hexagonal prism is truncated by blue $(10\bar{1}0)$ and purple (0001) faces.

Kinetic Monte-Carlo Simulations

To probe the termination effect on the growth speed of facets, we performed a kinetic Monte-Carlo simulation of epitaxial growth of a surface with alternating layers of A and B. As a toy model, we used a square lattice for the growth of a 160 × 160 unit cell. The bulk chemical potential of each layer was set to

$\Delta\mu$ = -0.30 k_BT, with an edge energy of γ_{edge} = 0.65 k_BT. The surface energy difference $\Delta\gamma_{A\text{-}B}$ was varied from 0 to 0.29 k_BT and the mean surface height (as a function of time) was recorded to generate a graph shown in Supplementary Fig. 5. This allowed us to calculate the mean growth rate of a system with different energy barriers. We recorded the mean time spent on low energy layers, discarding the results where multiple growth events occurred at the same time. A clear increase in the mean time is observed when $\Delta\gamma_{A\text{-}B}$ > 0.15. This indicates that beyond certain amount of $\Delta\gamma_{A\text{-}B}$, the epitaxial growth transitions from a monolayer to a multilayer growth (Fig. 5). In the case where a very low number of layers is grown for large $\Delta\gamma_{A\text{-}B}$ (2–3 layers), it generates a large confidence interval. For small values of $\Delta\gamma_{A\text{-}B}$, particle nucleation proceeds quickly and can be faster than the time it takes for a terrace to fully grow, yielding no plateau. These values are discarded from the fitting process. Confidence intervals for the mean surface growth is determined by splitting the mean surface height in 10 different bins and calculating their standard deviation.

As illustrated in Fig. 5d, at $\Delta\gamma_{A\text{-}B}$ = 0, the $\Delta\mu$ and γ_{edge} values considered in these simulations results in rough surface. For the Wulff equilibrium crystal structures observed in previous experiments[8,10], all colloids are equivalent to each other, and thus $\Delta\gamma_{A\text{-}B}$ = 0. Therefore, the simulation results displayed do not agree well with the experimental results. Although the parameters chosen in this study are not ideal values to model experiments, we will discuss several reasons why these parameters were chosen.

Based on the equations derived in the main text, for small values of $\Delta\gamma_{A\text{-}B}$ the mean growth velocity is predicted to be inversely proportional to $E_{A/B} + E_{B/A}$. For large values of $\Delta\gamma_{A\text{-}B}$, it should be inversely proportional to E_{BA}. These values can be directly correlated to the mean time between nucleation events shown in Fig. 5c; yet, a more dramatic change is observed for the mean time than the growth velocity. This is because multiple nucleation events are occurring within the time frame of a monolayer growth. For the expected layer-by-layer growth, these two curves should display stronger correlations.

Using either a smaller value of $\Delta\mu$ (in magnitude) or a greater value of γ_{edge} in the simulations leads to a smooth surface (Supplementary Fig. 6) as observed in previous experiments. However, in this case, the growth is significantly slowed, especially with large $\Delta\gamma_{A\text{-}B}$ and poor statistics, resulting in growth of only a few layers in the simulations. Nevertheless, Supplementary Fig. 6 illustrates that the calculations performed in this work exhibit an anticipated trend where the growth is dramatically slowed and the mean time between nucleation events increases. The full curve beyond $\Delta\gamma_{A\text{-}B}$ = 0.3 k_BT cannot be obtained.

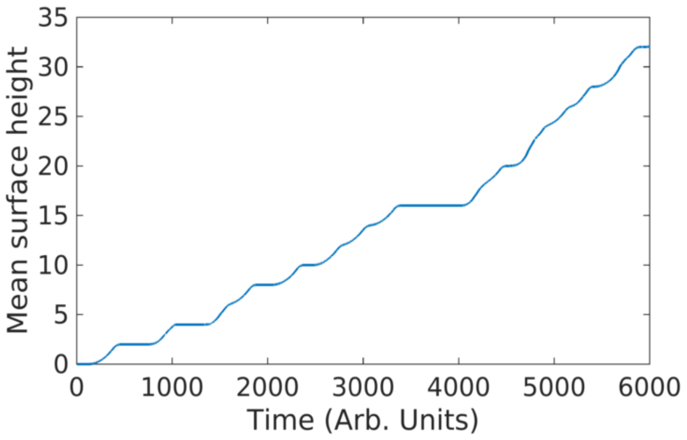

Supplementary Figure 5. Mean surface height of the thin film graphed as a function of time for the case $\Delta\gamma_{A\text{-}B}$ = 0.198 k_BT. Even (odd) number of monolayers correspond to low (high) energy surfaces.

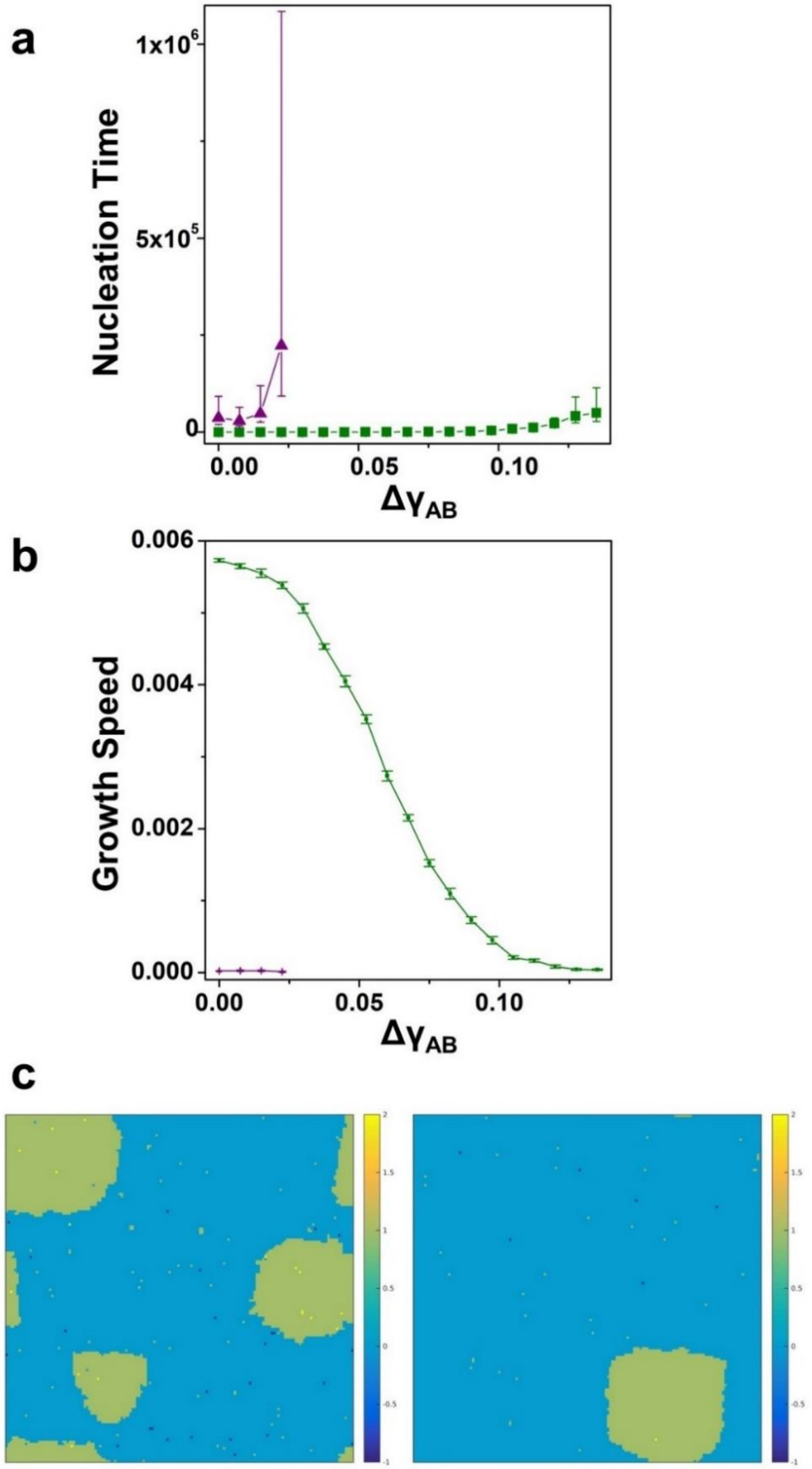

Supplementary Figure 6. a, Mean time between sequential nucleation events. **b,** Mean surface growth velocity. For both **a** and **b**, green points represent γ_{edge} = 0.80 k_BT and purple points represent γ_{edge} = 0.95 k_BT. **c,** Typical surface profile at $\Delta\gamma_{A\text{-}B}$ = 0 (left; γ_{edge} = 0.80 k_BT and right; γ_{edge} = 0.95 k_BT).

Supplementary Discussions

Hexagonal Prisms with Tunable *c/a* Ratios

Like atomic crystals, PAEs exhibit a well-defined, equilibrium interparticle distance that balances favorable attraction with unfavorable repulsion. Although atoms behave like hard spheres, a soft, polymeric DNA ligand shell around the rigid nanoparticle core allows one to engineer colloidal crystals with tunable lattice parameters. Typically, the PAE crystallization process is complicated by various types of interparticle interactions such as DNA hybridization attraction, steric repulsion between the DNA brushes, long-range electrostatic repulsion arising from the negatively charged PO_4^- groups of the DNA backbone, elastic repulsion, and repulsion from entropic effects due to counterions. It has recently been shown that the main thermodynamic contributor to lattice stability is the balance between attraction from DNA hybridization and repulsion due to excluded volume interactions between the DNA brushes[1]. Even though DNA is a polyanionic species, high salt concentration in which the PAEs are assembled effectively screens the long-range repulsion between the PAEs, which decays as a factor of $e^{-\kappa r}$ (κ is the inverse of the Debye length (charge screening distance) and r is the center-to-center distance). Therefore, one can assume that the short-range electrostatic repulsion should only be considered when the DNA brushes on the surface of particles are forced into close proximity. The Debye length, which decays as a factor of $I^{-1/2}$ (I is the salt concentration), should be integrated into the effective radius of the DNA strand when calculating the interaction potential from excluded volume repulsion[1]. With complementary PAEs, both attraction and repulsion govern their interactions whereas only repulsion exists between noncomplementary PAEs (*i.e.,* repulsion between like-charged ions, *e.g.,* A-A and B-B pairs). Therefore, by precisely tuning the attraction and repulsion between the PAEs, each lattice parameter in different dimensions (x, y, z) can be altered independently in the case of non-cubic lattices. For cubic lattices, a, b, and c are always equivalent to each other geometrically. The AB_2 superlattice is an example of non-cubic lattices where the lattice parameters, a (= b) and c, can be varied independently.

Colloidal crystals generated from DNA-modified building blocks are responsive to various external stimuli. Several strategies have been developed to alter the crystallization paths such as changing the DNA sequence, length, and number of oligonucleotides and varying salt concentration[3,4,11]. Depending on the method used, one can precisely tune the interaction between complementary PAEs or all the PAEs within the unit cell. For example, salt can be used to dynamically modulate superlattice structure because increasing salt results in a decrease in the Debye length, and thus the steric repulsion between the DNA brushes. Since the steric repulsion is experienced by both complementary and noncomplementary PAEs within the lattice when the DNA coronae are in contact, the overall lattice tends to compress in all dimensions. On the other hand, tuning the DNA bond strength mainly affects the interaction between PAEs that are interconnected *via* DNA hybridization events unless the length of the DNA bond is changed (*e.g.*, number of bases on DNA sticky end).

In addition to the data presented in the main text, we show that either changing the bond strength *via* DNA sticky end design or varying salt concentration can be used to tune the lattice parameters of the AB_2 lattices (Supplementary Table 7, Supplementary Fig. 8). Indeed, the SAXS data clearly reveal the change in crystal parameters. First, it is important to understand the type of interparticle interactions that are present in an AB_2 lattice. In Supplementary Fig. 7, there are twelve pairs of complementary PAEs, three pairs of noncomplementary (blue) PAEs along the *x,y*-plane, one pair of noncomplementary (blue) PAEs in the *z*-direction, and three pairs of noncomplementary (red) PAEs along the *x,y*-plane per unit cell.

Interactions between complementary and noncomplementary PAEs within a unit cell are denoted by green and red sticks, respectively (Supplementary Fig. 7). As stated in the main text, the change in molar ratio of PAEs added into the solution does not alter the crystal paths and results in the formation of same hexagonal prisms. Consistent with the SEM results, the resulting SAXS data show that all samples with the same combination of PAEs generated nanoscale architectures that exhibit similar lattice parameters (Supplementary Fig. 8 b, d; c, e, f; i, j). When the number of bases on the DNA sticky end is increased, the lattice parameter *a* increased whereas *c* either decreased or stayed constant (Supplementary Fig. 8). This can be attributed to stronger repulsive interactions between noncomplementary PAEs in the *x,y*-plane compared to the ones in the *z*-direction. For example, by simply evaluating the repulsion experienced by a PAE noted as "A" in Supplementary Fig. 7, one can clearly visualize that there are six neighboring noncomplementary PAEs along the *x,y*-plane (Supplementary Fig. 7a) compared to only two neighboring noncomplementary PAEs in the *z*-direction (top (omitted) and bottom) that are repulsive (Supplementary Fig. 7b). Therefore, it is more thermodynamically favorable to compress in the *z*-direction than along the *x,y*-plane. As the concentration of salt was increased from 1 to 2 M NaCl, the superlattices were compressed approximately 3% along the *x,y*-plane and 1% in the *z*-direction for all samples.

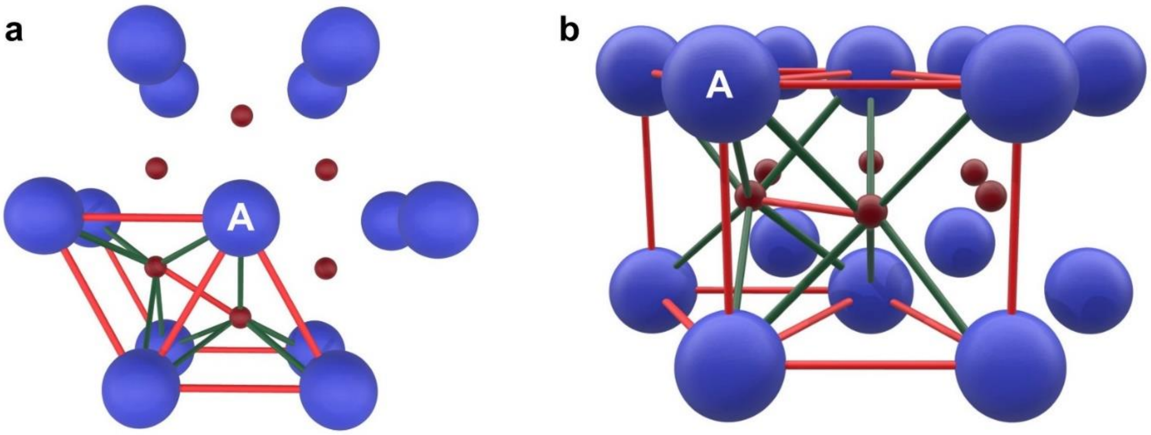

Supplementary Figure 7. Top-down (**a**) and side (**b**) views of the AB_2 superlattices. **a,** The PAE "A" has six neighboring like-PAEs (blue) along the *x,y*-plane that are repulsive. In addition, two red PAEs in the center of a unit cell interact *via* repulsion. **b,** The PAE "A" has two neighboring like-PAEs along the *z*-direction (a PAE above "A" is omitted) that are repulsive. Blue and red PAEs are connected *via* DNA hybridization interactions. Green and red sticks denote interactions between complementary and noncomplementary PAEs within a unit cell, respectively.

This section presents both the SAXS data used to characterize these lattices, the SEM images of hexagonal prisms, and the relevant assembly and crystal parameters for each sample synthesized in this work. The data below consist of: experimental SAXS data (black, solid line), modeled SAXS data (red, vertical lines), SEM images, and a model unit cell drawn to scale. The modeled data were generated using MATLAB as described in the Methods section. Modeled SAXS data are included as a reference for the experimental SAXS data. R_{NP} α and β values are the radii of the big and small inorganic nanoparticle cores, respectively. Linker # represents the type of linker strand that was hybridized onto DNA-functionalized nanoparticles (Supplementary Table 1). Values of β/α represent the estimated ratio between hydrodynamic radii of the PAEs, as calculated using Supplementary Eq. 1. In the case of particle molar ratio added in excess (greater than 1:2), the resulting slow cooled solution was red, indicative of excess PAEs that are not associated within the lattice. The interparticle distance is the center-to-center distance between two closest

neighboring PAEs that are directly linked with DNA (Supplementary Eq. 2). The lattice parameters are the edge lengths of the given unit cells, as calculated using the SAXS results and Supplementary Table 2. *c/a* is the ratio between the edge lengths in *x*- and *z*-directions.

Supplementary Table 7. Summary of Lattice Parameters of AB_2 Structures.

Particle Size Ratio / Stoichiometry	Salt Concentration (M)		*a* (nm)	*c* (nm)	Interparticle Distance (nm)
	1	2			
**0.80 / 1:2**		v	39.9	38.2	29.9
**0.66 / 1:2**	v		39.5	39.2	30.1
**0.66 / 1:2**		v	38.5	38.7	29.5
**0.66 / 1:3**	v		39.4	39.0	29.9
**0.66 / 1:3**		v	38.4	38.5	29.4
**0.66 / 1:6**	v		38.4	38.9	29.5
**0.67 / 1:2**		v	39.9	37.7	29.8
**0.67 / 1:6**		v	40.3	39.0	30.3
**0.73 / 1:2**	v		43.0	39.2	31.6
**0.74 / 1:3**		v	43.4	39.1	31.8

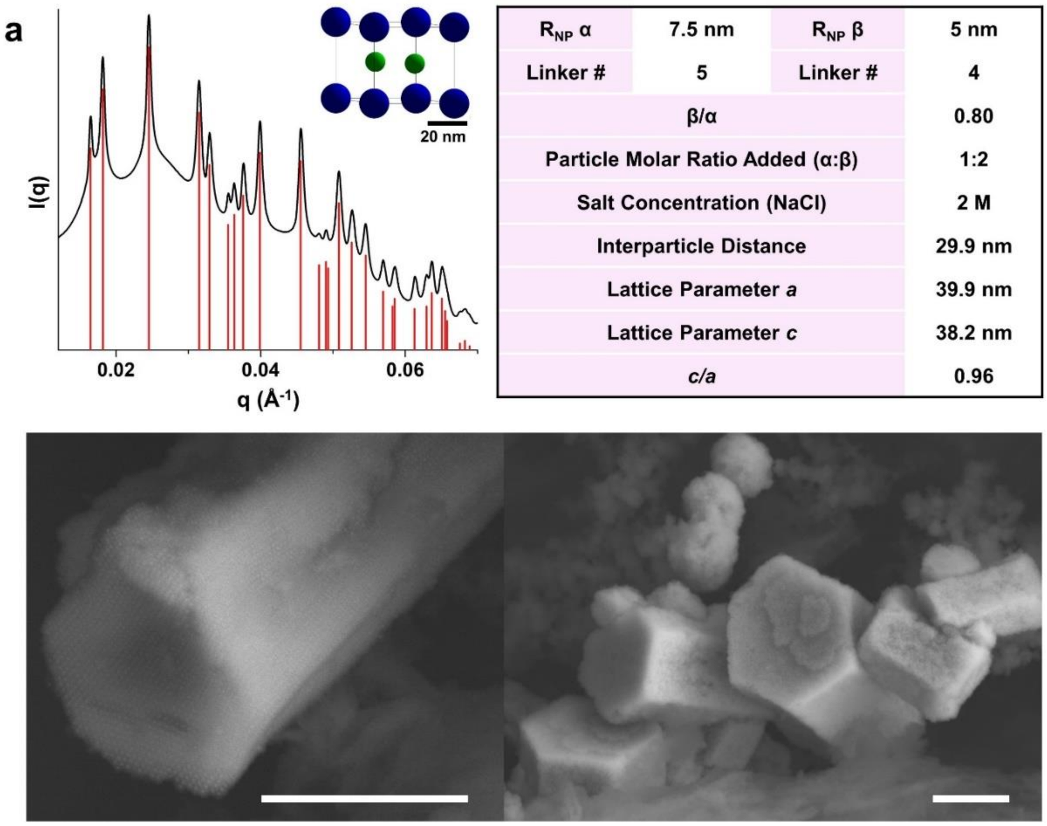

b

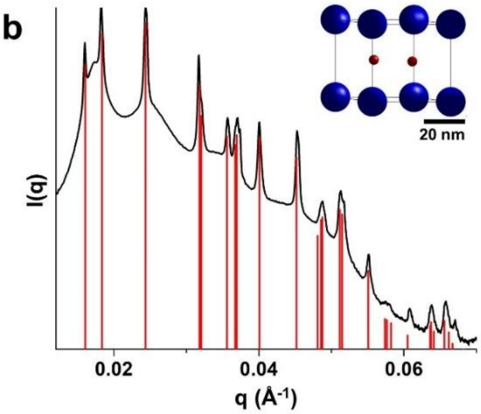

R_{NP} α	7.5 nm	R_{NP} β	2.5 nm
Linker #	5	Linker #	4
β/α			0.66
Particle Molar Ratio Added (α:β)			1:2
Salt Concentration (NaCl)			1 M
Interparticle Distance			30.1 nm
Lattice Parameter *a*			39.5 nm
Lattice Parameter *c*			39.2 nm
c/a			0.99

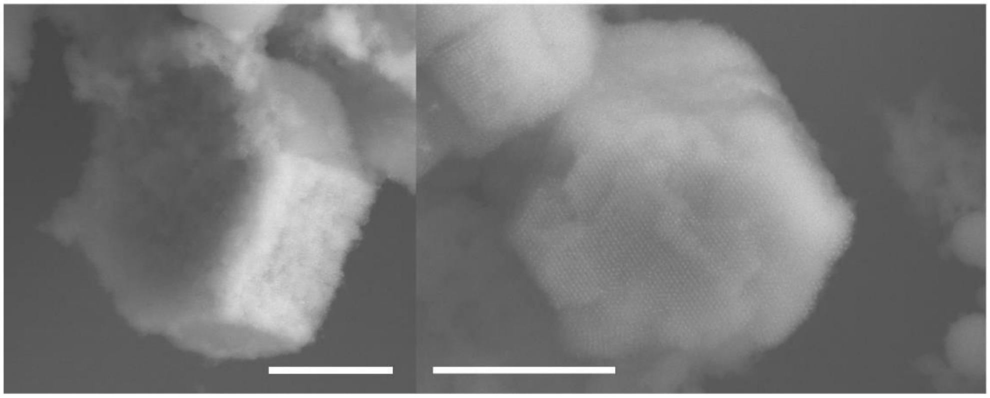

c

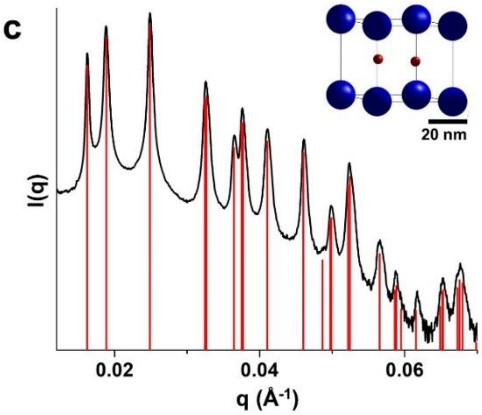

R_{NP} α	7.5 nm	R_{NP} β	2.5 nm
Linker #	5	Linker #	4
β/α			0.66
Particle Molar Ratio Added (α:β)			1:2
Salt Concentration (NaCl)			2 M
Interparticle Distance			29.5 nm
Lattice Parameter *a*			38.5 nm
Lattice Parameter *c*			38.7 nm
c/a			1

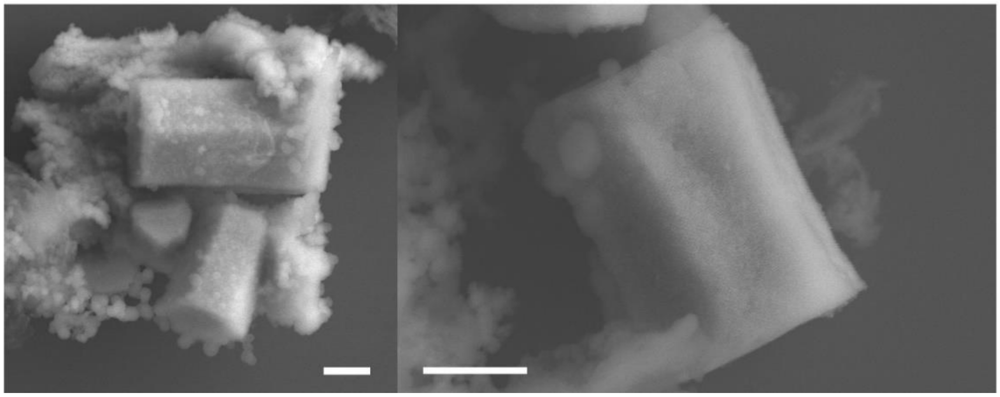

d

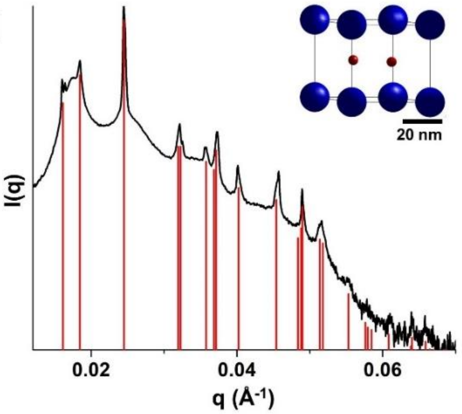

R_{NP} α	7.5 nm	R_{NP} β	2.5 nm
Linker #	5	Linker #	4
β/α			0.66
Particle Molar Ratio Added (α:β)			1:3
Salt Concentration (NaCl)			1 M
Interparticle Distance			29.9 nm
Lattice Parameter *a*			39.4 nm
Lattice Parameter *c*			39.0 nm
c/a			0.99

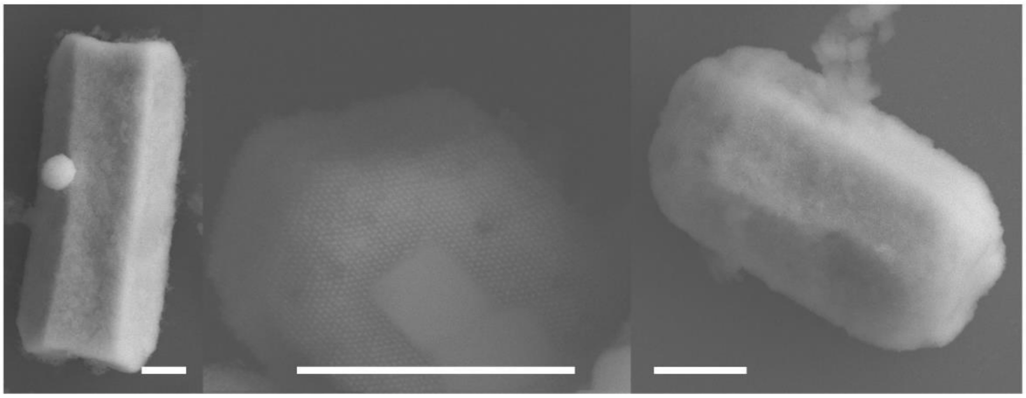

e

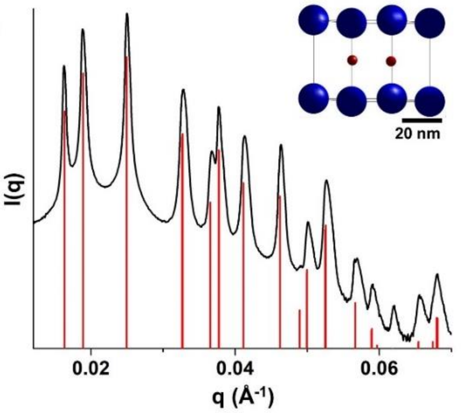

R_{NP} α	7.5 nm	R_{NP} β	2.5 nm
Linker #	5	Linker #	4
β/α			0.66
Particle Molar Ratio Added (α:β)			1:3
Salt Concentration (NaCl)			2 M
Interparticle Distance			29.4 nm
Lattice Parameter *a*			38.4 nm
Lattice Parameter *c*			38.5 nm
c/a			1

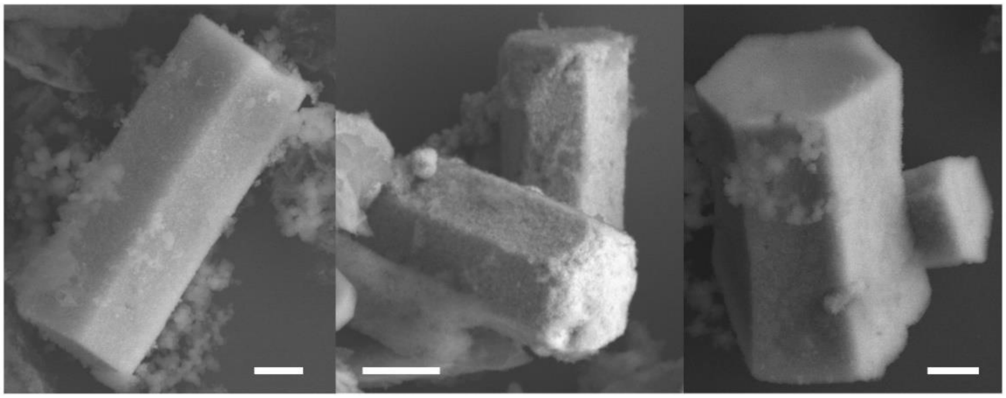

f

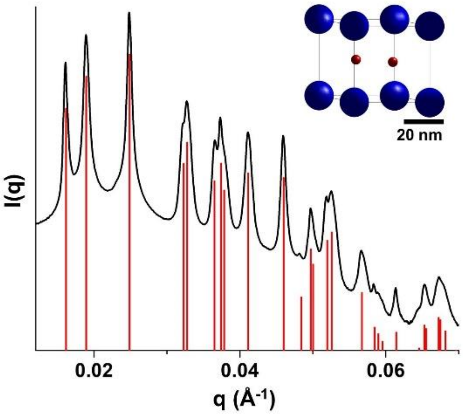

R_{NP} α	7.5 nm	R_{NP} β	2.5 nm
Linker #	5	Linker #	4
β/α			0.66
Particle Molar Ratio Added (α:β)			1:6
Salt Concentration (NaCl)			1 M
Interparticle Distance			29.5 nm
Lattice Parameter *a*			38.4 nm
Lattice Parameter *c*			38.9 nm
c/a			1

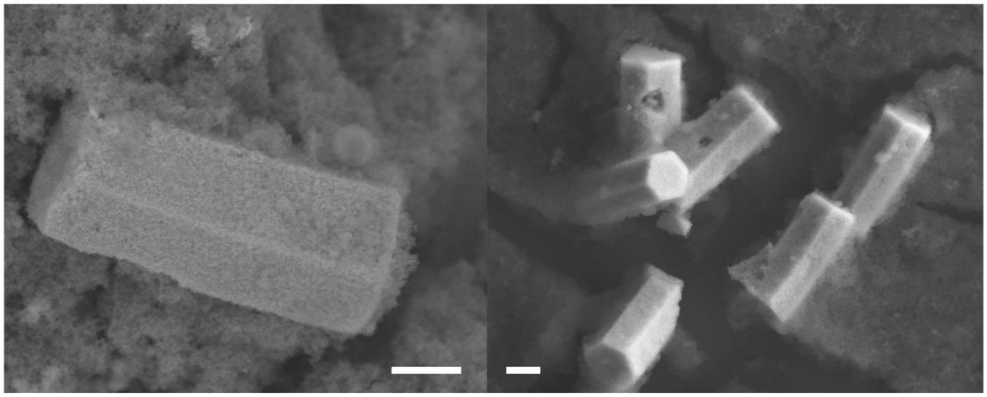

g

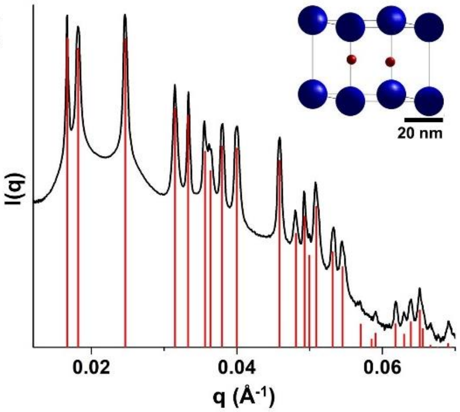

R_{NP} α	7.5 nm	R_{NP} β	2.5 nm
Linker #	7	Linker #	6
β/α			0.67
Particle Molar Ratio Added (α:β)			1:2
Salt Concentration (NaCl)			2 M
Interparticle Distance			29.8 nm
Lattice Parameter *a*			39.9 nm
Lattice Parameter *c*			37.7 nm
c/a			0.95

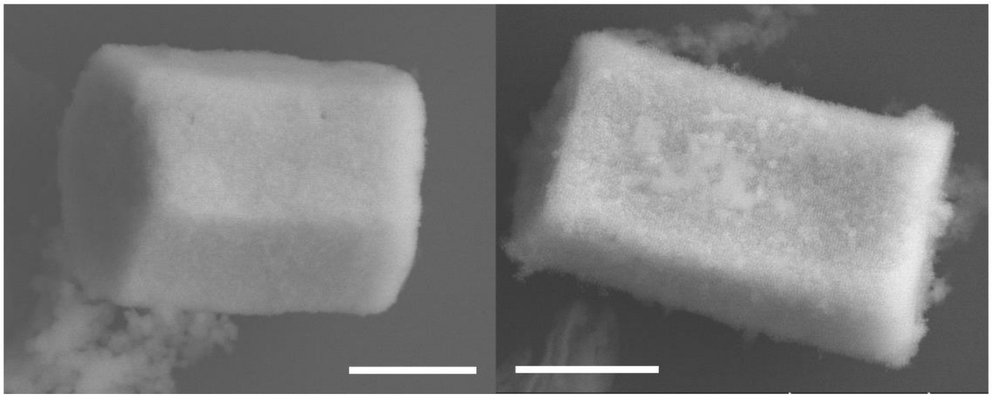

h

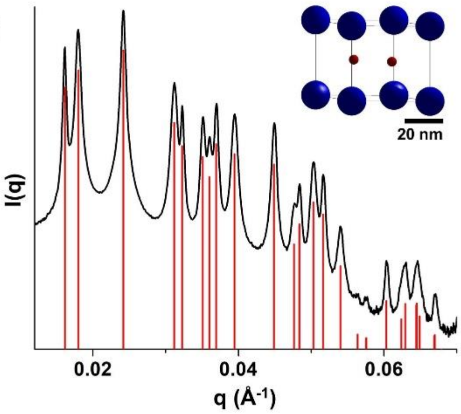

R_{NP} α	7.5 nm	R_{NP} β	2.5 nm
Linker #	7	Linker #	6
β/α			0.67
Particle Molar Ratio Added (α:β)			1:6
Salt Concentration (NaCl)			2 M
Interparticle Distance			30.3 nm
Lattice Parameter *a*			40.3 nm
Lattice Parameter *c*			39.0 nm
c/a			0.97

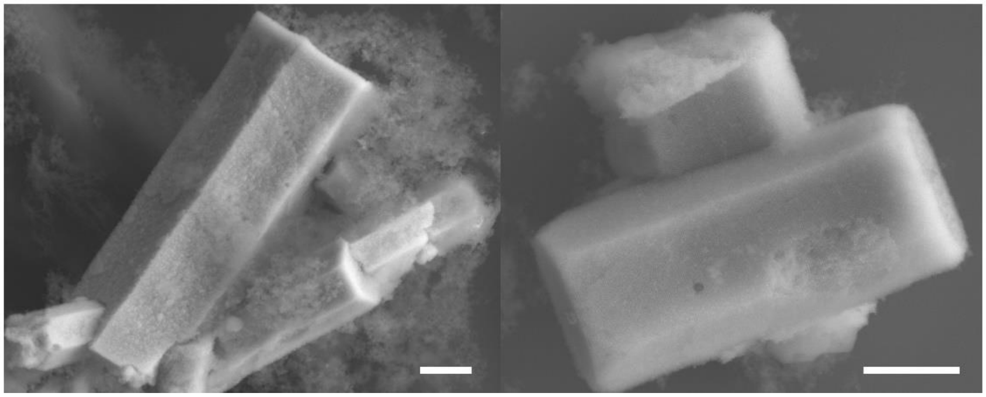

i

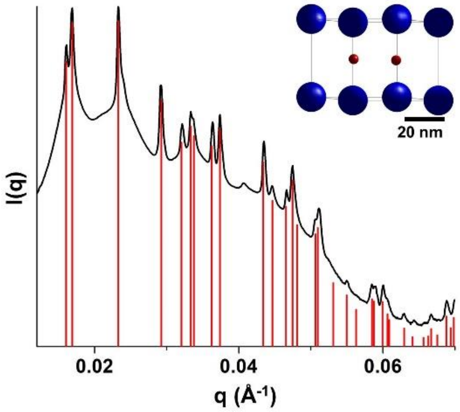

R_{NP} α	7.5 nm	R_{NP} β	2.5 nm
Linker #	5	Linker #	4
β/α			0.73
Particle Molar Ratio Added (α:β)			1:2
Salt Concentration (NaCl)			1 M
Interparticle Distance			31.6 nm
Lattice Parameter *a*			43.0 nm
Lattice Parameter *c*			39.2 nm
c/a			0.91

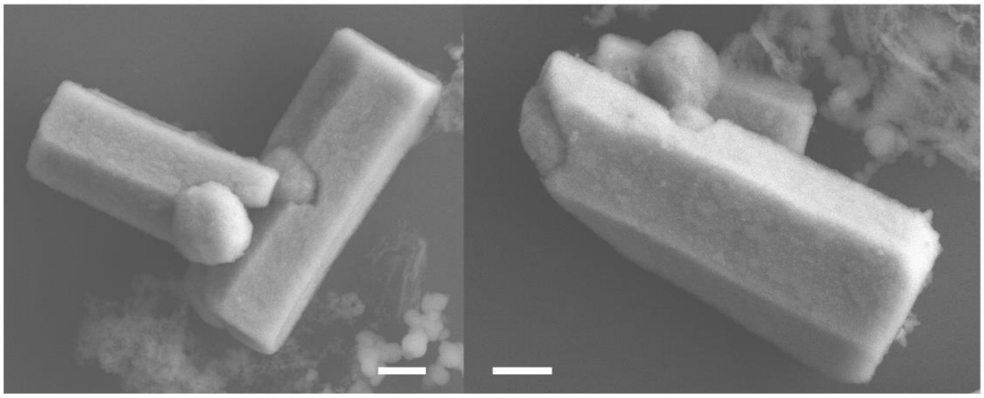

j

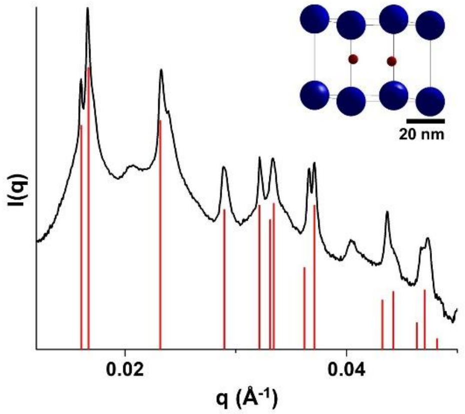

R_{NP} α	7.5 nm	R_{NP} β	2.5 nm
Linker #	5	Linker #	4
β/α			0.74
Particle Molar Ratio Added (α:β)			1:3
Salt Concentration (NaCl)			2 M
Interparticle Distance			31.8 nm
Lattice Parameter *a*			43.4 nm
Lattice Parameter *c*			39.1 nm
c/a			0.9

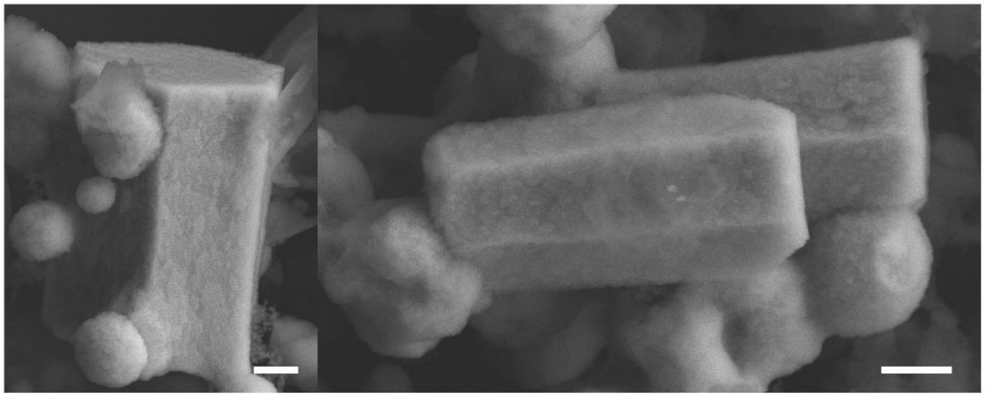

Supplementary Figure 8. a-j, SAXS pattern, SEM images, and relevant crystal parameters for AB_2 (isostructural with aluminum diboride) lattices assembled at (**a**) a *β*/*α* of 0.8, a molar ratio of particles added (*α*:*β*) in 1:2, and a salt concentration of 2 M, (**b**) a *β*/*α* of 0.66, an *α*:*β* of 1:2, and a salt concentration of 1 M, (**c**) a *β*/*α* of 0.66, an *α*:*β* of 1:2, and a salt concentration of 2 M, (**d**) a *β*/*α* of 0.66, an *α*:*β* of 1:3, and a salt concentration of 1 M, (**e**) a *β*/*α* of 0.66, an *α*:*β* of 1:3, and a salt concentration of 2 M, (**f**) a *β*/*α* of 0.66, an *α*:*β* of 1:6, and a salt concentration of 1 M, (**g**) a *β*/*α* of 0.67, an *α*:*β* of 1:2, and a salt concentration of 2 M, (**h**) a *β*/*α* of 0.67, an *α*:*β* of 1:6, and a salt concentration of 2 M, (**i**) a *β*/*α* of 0.73, an *α*:*β* of 1:2, and a salt concentration of 1 M, and (**j**) a *β*/*α* of 0.74, an *α*:*β* of 1:3, and a salt concentration of 2 M. A model unit cell in each data set is drawn to scale. Black lines in the model lattice denote the edges of the unit cell. Scale bars are 1 µm. Depending on the batch of particles, the lattice parameters may vary slightly, which we attribute it to different DNA loading or DNA linker loading on particles.

Additional Electron Microscopy Images

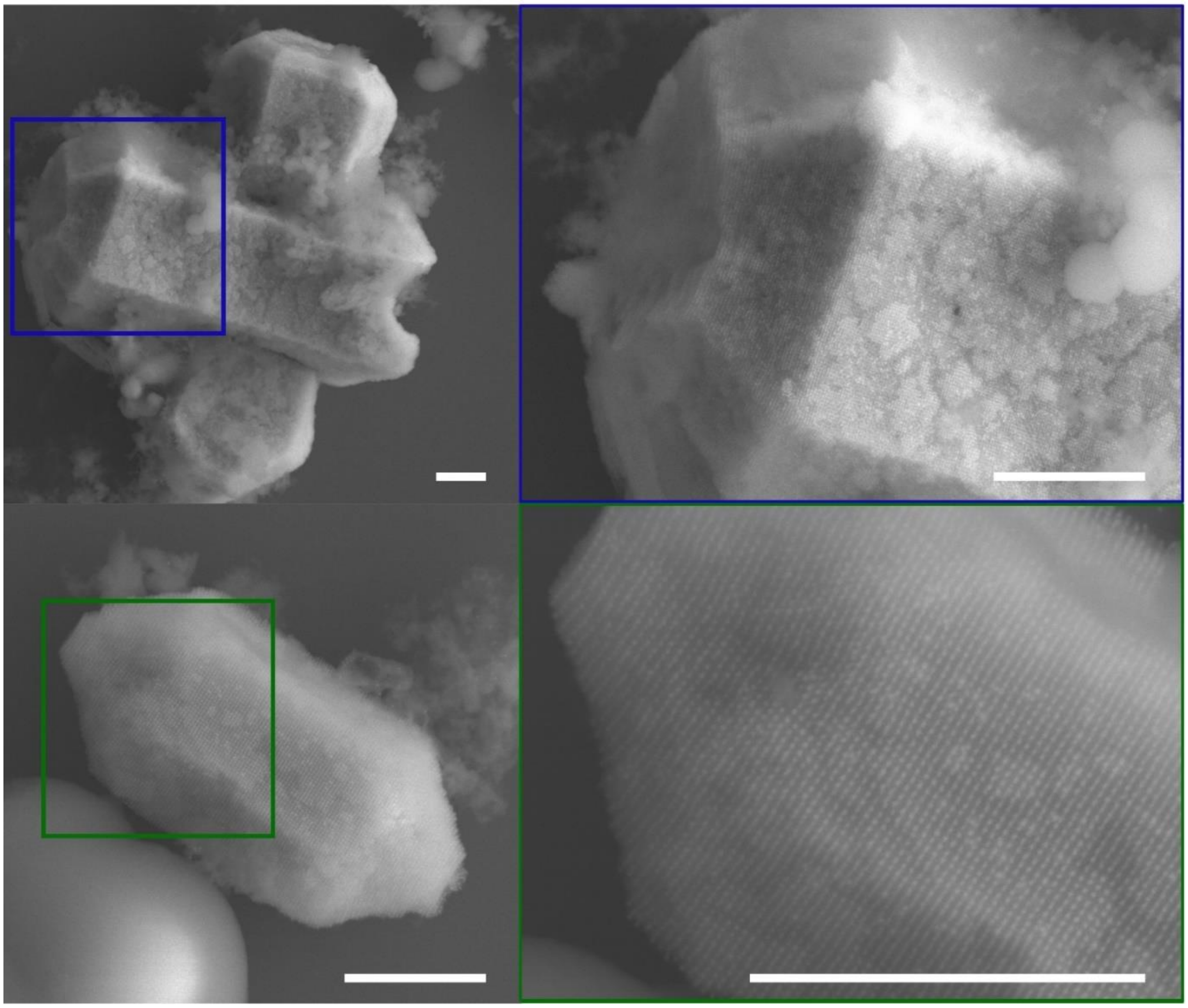

Supplementary Figure 9. SEM images of hexagonal prisms with noticeable shape deformations near the tips. These crystals are generated from superlattices with AlB_2 crystallographic symmetries. Based on geometrical analysis, new surfaces are exposing the $AB_2(11\bar{2}1)$ facet, which is another high surface energy facet. Scale bars are 1 μm.

Supplementary References

1 Seo, S. E., Li, T., Senesi, A. J., Mirkin, C. A. & Lee, B. The role of repulsion in colloidal crystal engineering with DNA. *J. Am. Chem. Soc.* **139**, 16528-16535 (2017).

2 Hurst, S. J., Lytton-Jean, A. K. & Mirkin, C. A. Maximizing DNA loading on a range of gold nanoparticle sizes. *Anal. Chem.* **78**, 8313-8318 (2006).

3 Wang, M. X. *et al.* Altering DNA-programmable colloidal crystallization paths by modulating particle repulsion. *Nano Lett.* **17**, 5126-5132 (2017).

4 Macfarlane, R. J. *et al.* Nanoparticle superlattice engineering with DNA. *Science* **334**, 204-208 (2011).

5 Li, T., Senesi, A. J. & Lee, B. Small angle X-ray scattering for nanoparticle research. *Chem. Rev.* **116**, 11128-11180 (2016).

6 Knorowski, C., Burleigh, S. & Travesset, A. Dynamics and statics of DNA-programmable nanoparticle self-assembly and crystallization. *Phys. Rev. Lett.* **106**, 215501 (2011).

7 Li, T. I., Sknepnek, R., Macfarlane, R. J., Mirkin, C. A. & Olvera de la Cruz, M. Modeling the crystallization of spherical nucleic acid nanoparticle conjugates with molecular dynamics simulations. *Nano Lett.* **12**, 2509-2514 (2012).

8 O'Brien, M. N., Lin, H.-X., Girard, M., Olvera de la Cruz, M. & Mirkin, C. A. Programming colloidal crystal habit with anisotropic nanoparticle building blocks and DNA bonds. *J. Am. Chem. Soc.* **138**, 14562-14565 (2016).

9 Zucker, R. V., Chatain, D., Dahmen, U., Hagège, S. & Carter, W. C. New software tools for the calculation and display of isolated and attached interfacial-energy minimizing particle shapes. *J. Mater. Sci.* **47**, 8290-8302 (2012).

10 Auyeung, E. *et al.* DNA-mediated nanoparticle crystallization into Wulff polyhedra. *Nature* **505**, 73-77 (2014).

11 Thaner, R. V. *et al.* Entropy-driven crystallization behavior in DNA-mediated nanoparticle assembly. *Nano Lett.* **15**, 5545-5551 (2015).
